# Clinical and Physiological Perspectives of β-Glucans: The Past, Present, and Future

**DOI:** 10.3390/ijms18091906

**Published:** 2017-09-05

**Authors:** Khawaja Muhammad Imran Bashir, Jae-Suk Choi

**Affiliations:** 1 Seafood Research Center, IACF, Silla University, Advanced Seafood Processing Complex #606, Wonyang-ro, Amnam-dong, Seo-gu, Busan 49277, Korea; imranagrarian3@gmail.com; 2 Major in Food Biotechnology, Division of Bioindustry, College of Medical and Life Sciences, Silla University, 140, Baegyang-daero 700 beon-gil, Sasang-gu, Busan 46958, Korea

**Keywords:** anti-obesity, anti-osteoporosis, antitumor, β-glucans, bioactive polysaccharides, immunomodulation

## Abstract

β-Glucans are a group of biologically-active fibers or polysaccharides from natural sources with proven medical significance. β-Glucans are known to have antitumor, anti-inflammatory, anti-obesity, anti-allergic, anti-osteoporotic, and immunomodulating activities. β-Glucans are natural bioactive compounds and can be taken orally, as a food supplement, or as part of a daily diet, and are considered safe to use. The medical significance and efficiency of β-glucans are confirmed in vitro, as well as using animal- and human-based clinical studies. However, systematic study on the clinical and physiological significance of β-glucans is scarce. In this review, we not only discuss the clinical and physiological importance of β-glucans, we also compare their biological activities through the existing in vitro and animal-based in vivo studies. This review provides extensive data on the clinical study of β-glucans.

## 1. Introduction

**Figure 1:**
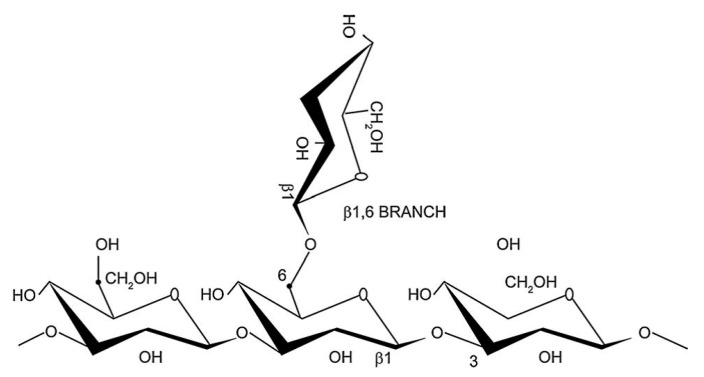
A linear 1,3 glycosidic chain of β-d-glucose monomers linked by a 1,6 glycosidic bond [11].

β-Glucans are groups of dietary fibers or polysaccharides composed of d-glucose monomers, linked by 1,3; 1,4 or 1,6 β-glycosidic bonds (Figure 1), and are naturally found in the cell wall of bacteria, fungi, algae, and higher crops, such as cereals. Highly-pure β-glucans are enzymatically extracted from the cell wall of yeast, fungi, seaweed, or grain seeds [1,2,3]. The biological and physiochemical properties of β-glucans strongly differ, depending on the source of extraction [4,5]. The degrees of purification, as well as the extraction method, also influence the physiological activity of β-glucans [6]. β-Glucans are generally divided into soluble and insoluble β-glucans, based on physiological properties [7]. In general, insoluble fibers decrease intestinal transit time as well as increase fecal bulk and the excretion of bile acids. However, soluble fibers slow glucose absorption and increase the total transit time by delaying gastric emptying [8]. Gel forming β-glucans are generally considered to be soluble β-glucans, including linear β-glucans (i.e., laminarin), high-molecular branched β-glucans (i.e., schizophyllan, grifolan, and scleroglucan), and chemically-modified particular β-glucans (i.e., phosphorylated or sulfonated β-glucans). However, most of these particular β-glucans are insoluble, such as yeast β-glucans [9]. The members of the first group are usually soluble in alkalies [10].

β-Glucans are a comparatively economical milling byproduct with proven health benefits. They are primarily isolated from the cell walls of yeast, fungi, and cereals, and the contents of β-glucans strongly depend on the environmental conditions [12,13,14,15]. Among cereals, the highest content of β-glucans per 100 g dry weight of barley and oat has been reported as 20 and 8 g, respectively. Other cereals also contain β-glucans, but in much lower amounts, as sorghum (6.2 g), rye (2.7 g), maize (1.7 g), triticale (1.2 g), wheat (1.0 g), durum wheat (0.6 g), and rice (0.13 g) [16]. Other sources of β-glucans include yeasts, such as *Saccharomyces cerevisiae*, mushrooms, such as Maitake and Shiitake, and seaweeds, such as *Laminaria* sp. [17,18]. The major β-glucans of clinical significance, their structures, and sources are listed in Table 1.

The health benefits of β-glucans have been extensively documented over the past two decades. β-glucans are allowed in several countries, including the United States of America, Canada, Finland, Sweden, China, Japan, and Korea, as potent immunological activators [19,20]. β-Glucans are used as a disease-preventing agent, as well as a part of anticancer or anti-inflammatory therapy. Among soluble fibers, β-glucans are the most commonly-consumed immunomodulator with strong anticancer, insulin resistance, anti-hypertension, and anti-obesity effects. β-Glucans are believed to stimulate the immune system, modulating humoral and cellular immunity, and thereby have beneficial effects in fighting infectious diseases, such as bacterial, viral, fungal, and parasitic diseases [21,22,23,24].

β-Glucans have proven characteristics in lowering blood total cholesterol and blood lipid profiles, as well as in maintaining body weight [25,26,27]. Kogan et al. demonstrated the potent inhibitory activity of β-glucans on lipid peroxidation, as well as the synergistic effects of β-glucans as antioxidant, antigenotoxic, and antimutagenic activities [28]. Daou and Zhang demonstrated the immune-stimulating activity of oat β-glucans by activating macrophages and increasing the amounts of immunoglobulin [29]. Murphy et al. reviewed the immune modulating effects of β-glucans and their subsequent benefits on infectious diseases and cancer [30]. Ooi and Liu reviewed the immunomodulating and anticancer effects of β-glucans from mushrooms, as well as the relationship of their structures and antitumor activities [31]. Antitumor mushroom polysaccharides, such as lentinan, schizophyllan, and krestin, have large markets in East Asian countries, including Japan, and Korea [31].

Jesenak et al. reviewed the impact of β-glucans on the treatment of allergic diseases [32], as well as the role of β-glucans in the management and prevention of respiratory tract infections [33]. Khoury et al. reviewed the capability of β-glucans in the prevention and treatment of metabolic syndrome, their underlying mechanisms of action, and their potential in food applications [34]. Chen and Raymond reported that β-glucans can mediate diabetes mellitus by controlling blood glucose levels and hypertension [35]. β-Glucans can reduce the risk factors associated with diabetes mellitus and benefit diabetes therapy. In addition, β-glucans can promote wound healing and alleviate ischemic heart injury. Hou et al. evaluated the effects of β-glucans on invasive fungal diseases [36]; they reported that a β-glucan assay is a useful screening tool with high sensitivity and specificity for discriminating between patients with and without invasive fungal diseases.

No adverse human effects have been reported following the consumption β-glucans, mainly from oat or barley [37]. The medical significance and effectiveness of β-glucans as antimicrobial, anticancer, anti-diabetic, and anti-hyperchloresterolemic polysaccharides have been reviewed [20,29,38]. However, systematic study of the clinical and physiological significance of β-glucans is scarce. Hence, there is a need to critically review the clinical and physiological aspects of β-glucans. In this review, we, not only discuss the clinical and physiological significance of β-glucans from selected studies, but we also compare with existing in vitro and animal-based in vivo studies. Our review provides extensive data on the clinical aspects of β-glucans.

## 2. Antitumor Effects of β-Glucans

The antitumor effects of β-glucans, extracted from different sources, have been extensively studied in vitro, as well as in animal-based in vivo studies; however, human-based clinical trials have rarely been reported. The antitumor effects of β-glucans are been listed in Table 2, Table 3 and Table 4 and are described below.

### 2.1. Antitumor Effects of β-Glucans—In Vitro Studies

The immunostimulatory effects of the extracellular and intracellular polysaccharide fractions of *Ganoderma lucidum* strain MZKI G97 were tested for the induction of interferon-γ (IFN-γ) and tumor necrosis factor-α (TNF-α) synthesis in primary cultures of human peripheral blood mononuclear cells, isolated from a buffy coat [113]. The TNF-α-inducing activity of *G. lucidum* fractions showed potential for use as a supporting therapy in cancer patients receiving chemotherapy and/or radiotherapy. The mechanisms of action of β-glucans from *Saccharomyces cerevisiae*, as an antigenotoxic, and anticlastogenic agent, as well as its capacity to preserve cell viability, were demonstrated by Oliveira et al. [114]; the study was carried out in the CHO-xrs5 and CHO-k1 cell lines. The tested doses of β-glucan (5–40 g/mL) did not show clastogenic effects; however, a chemoprotective effect was observed in CHO-k1 cell lines, whereas the yeast-derived β-glucan did not show a protective effect after treatment in repair-deficient CHO-xrs5 cell lines, which supports the involvement of bioantimutagenesis. Neither a genotoxic nor an antigenotoxic effect were observed in the CHO-k1 cell lines, however, yeast-derived β-glucan preserved cell viability in both cell lines.

The biological activities of β-glucans differ in terms of their sources and structures. Chan et al. compared the immunological effects of β-glucans from mushroom and barley, and the response of human dendritic cells to the isolated glucans [115]. β-glucans from different sources showed different immune potencies and effects on human immune cells, including dendritic cells. Yeast-derived particulate β-glucan (p-β-glucan) has the ability to activate macrophages and dendritic cells via the dectin-1 pathway [87]. Activated dendritic cells, by p-β-glucan, promoted Th1 and cytotoxic T-lymphocyte priming and differentiation. In an animal-based model and in vitro studies, yeast-derived p-β-glucan revealed significant antitumor immune responses. Yeast-derived p-β-glucan, alone, had no therapeutic effect, but significantly augmented antitumor monoclonal antibody-mediated therapeutic efficiency via the complement activation pathway. Qi et al. reported that the yeast-derived soluble p-β-glucan could be used as an adjuvant in antibody-mediated tumor therapy [87].

The immunostimulatory and antitumor activities of β-glucans (IS-2) purified from mutated *S. cerevisiae* were investigated by Yoon et al. [88]. IS-2 significantly inhibited lung metastasis in B16-BL6 melanoma and colon 26-M3.1 carcinoma cells, as well as in CDF1 mice. The survival time of tumor-bearing mice was prolonged when pretreated with IS-2, two days before tumor inoculation. IS-2 enhanced splenocyte proliferating activity during in vitro cytotoxicity analysis and produced various cytokines, such s IL-12, IFN-γ, and IL-1β. IS-2 also induced the antitumor activity of the peritoneal macrophages against colon 26-M3.1 cells and supported natural killer cell cytotoxicity against Yac-1 tumor cells. IS-2 β-glucan inhibited tumor metastasis by activating natural killer cells and macrophages.

The association of Th17-inducing activities with notch ligand expression was studied by Higashi et al. [116]. A mixed lymphocyte reaction was induced by co-culturing human monoclonal-dendritic cells (Mo-DCs) with HLA-DR-non shared allogeneic CD4+ naive T cells and curdlan as an adjuvant. The expression of notch ligand in THP-1 cells and Mo-DCs were evaluated using the enzyme-linked immunosorbent assay (ELISA), as well as RT-PCR, for the presence of interleukins (IL-17, IL-5), and IFN-γ. Curdlan induced DC-mediated Th17 differentiation and upregulated Jagged1 mRNA expression in THP-1 and Mo-DCs. Higashi et al. reported that bacterial β-glucans (curdlan) have the ability to induce human DC-mediated Th17 polarization, which shows the tumor suppressing activities of curdlan [116].

The antitumor activities of yeast-, and fungi-derived insoluble β-glucans have been widely recognized, but the insolubility of these compounds creates several problems, especially in topical formulations. Furthermore, the high-molecular weight and high-viscosity of oat-derived β-glucans restrict their application. Choromanska et al. studied the antitumor activities of low-molecular-weight 1,3;1,4-β-glucan derived from oat in normal and cancerous cells [117]. The low-molecular-weight β-glucan from oat significantly decreased cancer cell viability, while, for normal cells, it was non-toxic. This study showed the strong antitumor potential of low-molecular-weight β-glucan from oat, which showed no toxicity to normal cells.

### 2.2. Antitumor Effects of β-Glucans—Animal Studies

The antitumor activities and physicochemical properties of a fungal β-glucan (OL-2), isolated from *Omphalia lapidescens*, were examined by Ohno et al. [122]. OL-2 displayed sharp signals on a nuclear magnetic resonance spectrum and a significant change in λ*_max_* to a longer wavelength, which was observed using Congo red assay. OL-2 showed significant antitumor activity against the ascites form of Sarcoma-180 and MH-134. However, it showed no or low antitumor activity against the solid form of Sarcoma-180. Saito et al. reported that the antitumor activity of OL-2 is related to β-linked branching and it showed the potent antitumor effect of OL-2 against the solid form of Sarcoma-180 in imprinting control region (ICR) mice [123]. The water-soluble β-glucans (H-3-B, S-H-3-B) isolated from a hot-water extract of *Cryptoporus volvatus* also showed significant antitumor activities against Sarcoma-180 tumor [65]. Leung et al. investigated the antitumor activities of an alkaline-soluble β-glucan, isolated from the cell wall of *Flammulina velutipes* against Sarcoma-180 mice [118]. β-glucan was non-toxic to a brine shrimp assay. It did not show antitumor activity in vitro, but, when injected into mice, it triggered proliferation of splenic lymphocytes and showed vascular dilation and a hemorrhage response.

The antitumor effects of polysaccharide fractions prepared from hot water extracts of an edible mushroom, *Sparasis crispa*, against the solid form of Sarcoma-180 in ICR mice were studied by Ohno et al. [124]. In another study, Ohno and colleagues measured the antitumor activity of polysaccharide fractions from hot water extracts of *Agaricus blazei* against the solid form of Sarcoma-180 tumor in ICR mice [125]. The *S. crispa* fractions showed enhanced hematopoietic response to cyclophosphamide-induced leukopenic response in mice. Ebina and Fujimiya studied the antitumor effects of extracts from *A. blazei* Murill in a double-grafted tumor system comprising BALB/c mice and Meth-A tumor cells [126]. Intratumoral administration of ethanol, water-ethanol, and ammonium oxalate soluble fractions showed inhibited tumor growth of Meth-A tumor. Significant macrophage chemotactic factor activity, as well as serum levels of immunosuppressive acidic protein, were observed in the culture media from the tumor tissues.

Antitumor monoclonal antibodies (mAb) are known for their applicability in tumor therapy. mAb binds to the tumor and activates components by coating tumors with inactivated C3b (iC3b). Hong et al. investigated the effects of yeast β-glucan as an adjuvant for antitumor mAb therapy in C3 or CR3 deficient mice [119]. The yeast β-glucan degraded in the bone marrow into smaller soluble β-glucan fragments that were taken up by CR3 of the marginated granulocytes, and these β-glucans inhibited the growth of iC3b-opsonized tumor cells. Driscoll et al. compared the therapeutic efficacy of various sources of β-glucans [127]. The yeast β-glucan, in combination with anti-tumor mAb, resulted in significantly smaller tumor sizes and revealed an enhanced long-term survival compared to mAb alone or β-glucan extracts from other sources, such as mushrooms. Cytokine production was markedly decreased in dendritic cells (DCs), and MyD88-deficient macrophages, but not in CR3-deficient mice. The yeast β-glucan demonstrated much stronger adjuvant activity compared to mushroom β-glucan extracts in tumor therapy.

The chemoprevention of tumors is linked with the induction of xenobiotic metabolizing enzymes. Since the cytochromes are associated with metabolizing certain pro-carcinogens to their ultimate forms, the prevention of cancer can be achieved by down regulation of cytochromes via food-grade additives. Okamoto et al. reported that lentinan suppressed hepatic CYPIAs expression through the production of tumor necrosis factor-α and caused an increase in the DNA-binding activity of nuclear factor-κB [128]. Zhang et al. estimated the antitumor effects of polysaccharides (L-FV-IB) isolated from water extracts of *Lentinus edodes* against the solid form of Sarcoma-180 tumor [120]. L-FV-IB also showed significant antitumor activities in both in vitro and in vivo studies. Vetvicka and Yvin investigated the antitumor effects of phycarine isolated from *Laminaria digitate* [121]. Phycarine significantly stimulated phagocytic activity, as well as potentiated the synthesis and release of TNF-α, IL-6, and IL-1. In addition, it increased NK cell-mediated killing of tumor cells under both in vitro and in vivo studies. 

### 2.3. Antitumor Effects of β-Glucans—Clinical Studies

The therapeutic efficacy of fungal 1,3;1,6-β-glucan in patients with cancer, as well as in adjunctive therapy in patients receiving chemotherapy to suppress hematopoiesis, was studied by Weitberg et al. [129]. In this study, patients with advanced malignancies receiving chemotherapy were given β-glucan preparation and were monitored for tolerability and effect on hematopoiesis. The study showed that fungal β-glucan was well tolerated in cancer patients receiving chemotherapy, and it might have a beneficial effect on hematopoiesis in cancer patients. Ostadrahimi et al. studied the effect of yeast β-glucan on the quality of life in women with breast cancer undergoing chemotherapy [130]. The study was conducted on women with breast carcinoma and the patients were given 10-mg capsules of commercial yeast β-glucan, daily, for 21 days. The findings of these studies suggested that β-glucans might be useful as an adjuvant during chemotherapy to improve the quality of life of patients with breast cancer.

## 3. Immunomodulating Effects of β-Glucans

β-Glucans possess strong immune-modulatory activities, which have been proved by in vitro, as well as by animal- and human-based clinical trials. The immunomodulating effects of β-glucans are listed in Table 5, Table 6 and Table 7 and are described below.

### 3.1. Immunomodulating Effects of β-Glucans—In Vitro Studies

The immunomodulatory activities of yeast-derived cerevan on rat thymocytes were evaluated by Sandula et al. [90]. Cerevan showed higher stimulation indices compared to zymosan (a known immunomodulating β-glucan). Wakshull et al. investigated the immune-modulating and antimicrobial activity of yeast-derived β-glucan (PGG-glucan) in different human and murine cell line models [131]. PGG-glucan significantly enhanced the oxidative burst response of blood leukocytes and increased leukocyte microbial activity. The study shows that PGG-glucan is beneficial in enhancing neutrophil antimicrobial effects.

The effect of *Ganoderma lucidum*-derived polysaccharide-glucan (PS-G) on human monocyte-derived dendritic cells (DCs) was studied by Lin et al. [132]. It enhanced cell-surface expression of human DCs, leukocyte antigen, and interleukin. In addition, PS-G resulted in enhanced T cell-stimulatory capacity and increased secretion of interferons and interleukin-10. This study suggested that PS-G has the potential to regulate immune responses by regulating the activation and maturation of immature dendritic cells. Chaung et al. also reported that a synthetic particulate-β-glucan (p-β-glucan) significantly enhanced cell activity and phagocytosis in porcine alveolar and dendritic cells [133].

Chanput et al. compared the immunological aspects of β-glucans from different sources (oat, barley, and mushroom) on phorbol myristate acetate differentiated THP-1 macrophages [134]. All the tested β-glucans slightly upregulated inflammation-related gene expression, but the expression intensity and patterns differed. It was concluded that β-glucans from different sources show varying levels of immunomodulatory properties. Bobadilla et al. investigated the immunostimulatory properties of seaweed-derived water-soluble β-1,3/1,6-β-glucan on mouse cells [135]. The algae-derived β-glucan showed no adverse effects on the survival of cells and showed an increase in activated CD19^+^ B lymphocytes.

### 3.2. Immunomodulating Effects of β-Glucans—Animal Studies

The mitogenic and colony-stimulating factor (CSF)-inducing activity of β-glucan polysaccharide fractions, extracted from *Dictyophora indusiata* FISCH, were reported by Hara et al. [49]. The β-glucan polysaccharide fraction (T-4-N) showed mitogenic and CSF-inducing activity. Sakurai et al. investigated the effect of SSG-glucan isolated from *Sclerotinia sclerotiorum* on alveolar macrophage activities of CDF1 mice [137]. SSG-glucan, at a concentration of 80 mg/kg, showed increased lysosomal enzyme activity of alveolar macrophages. An increase in phagocytic activity and interleukin-1 production was also observed. The study proved that SSG-glucan can activate murine alveolar macrophage, both quantitatively, as well as qualitatively.

Di Luzio et al. investigated the immune-stimulating effect of yeast-derived β-glucan in A/J and C57BL/6 mice [138]. Significant reductions in the growth of syngeneic anaplastic mammary carcinoma and melanoma B16, and increased survival rates of mice with subcutaneous tumor implants, were observed. Yeast β-glucan decreased renal necrosis in *Staphylococcus aureus*-challenged mice. Yeast-derived soluble β-glucan showed significant antitumor and anti-staphylococcal activity.

Reynolds et al. investigated host-resistance to infectious-disease response of yeast β-glucan in mice, rats, and healthy cynomolgus male and female monkeys (*Macaca fascicularis*) [139]. The pre-infection administration of yeast β-glucan significantly enhanced the survival of mice against Venezuelan equine encephalomyelitis (VEE) or Raft valley fever virus and *Pseudomonas pseudomallei*. However, post-infection administration did not enhance the survival of mice. Similarly, pre-infection intravenous administration of β-glucan significantly increased resistance to virulent *Francisella tularensis*. A combined dose of yeast β-glucan and the VEE vaccine showed higher resistance to homologous virus challenge. A similar effect of combined doses was observed in cynomolgus monkeys. This study reported the adjuvant effect of yeast β-glucan in treating infectious diseases.

Hotta et al. investigated the antiviral effects of fungal polysaccharide schizophyllan in mice infected with the lethal Sendai virus [140]. Both oral and intraperitoneal administrations of schizophyllan were effective against the Sendai virus, and significantly inhibited virus infection and spread in lungs. Furthermore, schizophyllan accelerated protective immunity when administered together with low doses of a live Sendai virus vaccine. This study suggested the protective and adjuvant immune response of schizophyllan when administered together with a live Sendai virus vaccine. Kaiser and Kernodle reported the enhanced infection-preventing activity of PGG-glucan when used together with antibiotics (Cefazolin) in guinea pigs inoculated with *Staphylococcus* bacteria [141]. Guinea pigs receiving PGG-glucan and Cefazolin showed increased infection-prevention compared to those receiving Cefazolin or PGG-glucan alone. This study showed the synergistic effects of PGG-glucan and Cefazolin in preventing staphylococcal wound infection.

The immunostimulatory effects of oat-derived β-glucan formulations on *Eimeria vermiformis* disease resistance in C57BL/6 mice were studied by Yun et al. [142]. The administration of oat-derived β-glucan resulted in reduced fecal oocyst shedding, higher levels of total serum immunoglobulins and antigen-specific immunoglobulins compared to the non-treated control. Oat-derived β-glucan upregulated immune response with enhanced resistance to *Eimeria coccidiosis* in mice. Yeast β-glucan (WGP), as a prophylactic treatment, significantly reduced mortality due to anthrax infection, as well as inhibited the growth of cancer cells in mice [136]. Hetland et al. investigated the antimicrobial effects of SSG-glucan isolated from *Sclerotinia sclerotiorum* in six-weeks old female inbred, specific-pathogen-free NIH/OlaHsd mice [143]. SSG-glucan showed a significant dose-dependent protective effect against *Streptococcus pneumoniae* type 4 and 6B. The study showed that SSG-glucan can be used in the treatment of pneumococcal infection in mice.

Hasegawa et al. studied the immunomodulatory effects of β-glucan formulations of extracts from *Sparassis scispa* [144]. SC-glucans showed reduced tumor size of Sarcoma-180 tumor and prolonged survival of mice. In addition, blood IgE levels, and the scratching index, were decreased, while human natural killer cell cytotoxicity was enhanced. The SC-glucan in this study promoted a shift in the Th1/Th2 balance towards Th1-dominant immunity. Methotrexate is widely used in the treatment of malignant tumors and rheumatic disorders. However, its efficiency is often limited by severe side effects and toxic sequelae. Sener et al. investigated the protective effects of yeast β-glucan in methotrexate-induced toxicity [145]. The application of yeast β-glucan eradicated the depletion of tissue glutathione, and inhibited an increase in tissue malondialdehyde, myeloperoxidase activity, and collagen contents and suppressed tissue damage. In addition, yeast β-glucan inhibited leukocyte apoptosis and cell death. This study showed that yeast β-glucan may be beneficial in alleviating leukocyte apoptosis and oxidative tissue injury.

Shim et al. investigated the immunostimulating activities of β-glucans, isolated from *Agrobacterium* sp., on various cancer cell lines, as well in ICR mice [95]. An induction effect of IFN-γ and cytokines, as well as the adjuvant effect on antibody production, were observed. Vetvicka and Vetvickova investigated the immunological and pharmacological effects of β-glucans from different sources, including yeast, fungi, and cereals, in eight-week old female BALB/c mice [6]. The yeast-derived β-glucan (glucan #300) significantly stimulated the production of IL-2 by mouse splenocytes and phagocytosis of peripheral blood leukocytes. Furthermore, yeast-derived β-glucan significantly lowered blood sugar and cholesterol levels in mice. The remaining tested β-glucans showed marginal immunological activity. This study strongly supported the concept that the immunological effects of β-glucans depend on the source and method of extraction.

Vetvicka and colleagues studied the immunostimulatory effects of algae-derived β-glucans (phycarine) [103]. Phycarine showed significant stimulation of phagocytosis in peripheral blood cells and helped in Lewis lung carcinoma chemotherapy. The strong immunostimulatory effects of phycarine on experimentally-induced leucopenia were observed. A majority of phycarine was detected in the gastrointestinal tract, thus supporting the feasibility of using it in the treatment of gastrointestinal diseases. Later, Vetvicka’s group compared the immune-stimulating effects of β-glucans from different sources. Yeast-derived β-glucan (Βetamune) significantly stimulated phagocytosis and showed an increase in the synthesis and release of interleukins (IL-1, 2, 4, 6, 8, and 13), as well as tumor necrosis factor-α [146]. Vetvicka and Vancikova compared the stress-related immunosuppressing effects of β-glucans, extracted from different sources, in mice [147]. All the tested β-glucans showed the capability of inhibiting cold-stress-related inhibition; one of the β-glucan fractions (glucan #300) was able to retain phagocytosis at a normal level. In addition, glucan #300 inhibited the increase in stress-related corticosterone and retained IL-6 and IL-12 levels above those of the control.

McCormack et al. investigated the chemoimmunostimulatory properties of lentinan, extracted from *Lentula edodes*, in male BN/RijHsd rats [148]. A significant increase in weight gains, monocytes, blood cells, circulatory cytotoxic T-cells, and a reduction in anti-inflammatory cytokines IL-4, IL-6, and IL-10 were observed. The combined effects of lentinan in acute myeloid leukemia chemotherapy with cytarabine and idarubicin enhanced the average survival of rats. Mallick et al. explored the immunomodulatory properties of polysaccharide glucan formulations, prepared using the hot-alkaline extracts of *Astraeus hygrometricus* [149]. A significant increase in the production of interleukin-1 and nitric oxide, as well an increase in phagocytotic potential, were observed. The extracted polysaccharide glucan also showed increased activation of natural killer cells and the proliferation of splenocytes. This study showed no toxic effects on the tested organisms, which suggests that the extracted polysaccharide glucan from *A. hygrometricus* could be effectively used as an immunomodulatory agent.

Sugiyama et al. investigated the suppressive effects of paramylon, extracted from *Euglena gracilis* Z., on the development of atopic dermatitis-like skin lesions in NC/Nga mice [150]. Paramylon significantly inhibited the development of atopic dermatitis-like skin lesions with no adverse effects on weight loss. This study suggested that paramylon could be used as an alternative therapy to treat atopic dermatitis. Chang et al. investigated the immunopharmacological effects of β-glucan isolated from *Paenibacillus polymyxa* JB115 on 5–6-week-old Sprague-Dawley rats [151]. The isolated β-glucan showed a significant increase in red blood cell count, white blood cell count, hemoglobin, and thrombocytes, in male rats, whereas, no marked changes were observed in female rats. No adverse effects on general condition, growth, behavior, water, and feed consumption were observed. The β-glucan isolated from *P. polymyxa* demonstrated no toxic effects in rats, suggesting the strong immunostimulatory effect of *P. polymyxa* derived β-glucans.

The influence of a purified β-glucan preparation on canine atopy in dogs was studied by Beynen et al. [152]. The effects of β-glucan were studied in a double-blind, placebo-controlled trial. The dogs received 800 ppm of β-glucan, daily, for a period of eight weeks and the clinical signs (scaling, redness, thickness, itching, and stripping of skin) of atopic dermatitis were evaluated. The β-glucan-fed dogs showed significant improvements in the overall index of improvement of atopic dermatitis compared to the control. Hong et al. investigated the exercise-induced stress response of β-glucan on the expression of oncogenes (*c-Jun*, *c-Fos*) in male Sprague Dawley rats [153]. An enhanced expression of *c-Jun*, and *c-Fos* in the dentate gyrus, dorsal raphe and hypothalamus of rats, after exhaustive treadmill running, was observed. An increase in exhaustion time and suppression of exercise-induced increments of oncogene expression shows that β-glucan administration exerted an alleviating effect on exercise-induced stress in rats. These animal-based studies strongly suggested that β-glucans could serve as a good candidate as an immune-modulating agent.

### 3.3. Immunomodulating Effects of β-Glucans—Clinical Studies

The immune-modulatory effects of PGG-glucan in high-risk patients, undergoing major abdominal or thoracic surgery, were studied by Babineau et al. [154]. An international multicenter, randomized, double-blind, placebo-controlled study showed a dose-dependent response of PGG-glucan against postoperative infections. β-Glucans are suggested to play an important role in the development of respiratory and organic dust-related diseases. The anti-fungal immunostimulatory response of β-1,3 poly-glucose in patients infected with *Paracoccidioides brasiliensis* was studied by Meira et al. [155]. A significant reduction in erythrocyte sedimentation rate and serum antibody levels were observed; however, higher serum levels of tumor necrosis factor were observed. In addition, patients in post-operation treatment groups showed a positive reaction in the phytohemagglutinin skin test, as well an increase in CD4^+^ T lymphocytes. This study suggests that patients who received β-glucans showed stronger and more favorable responses to therapy.

Holck et al. studied the potential of yeast- and fungi-derived β-glucans to improve immune response to inflammatory and allergic diseases [156]. Twenty-eight- to fifty-six-year-old healthy volunteers, and patients allergic to house dust mites, were treated with curdlan, laminarin, scleroglucan, and pustulan. All the tested β-glucans showed enhancement of anti-immunoglobulin E (IgE) mediated histamine release. Sarinho et al. studied the immunomodulatory effects of yeast-derived β-glucans against asthma and other allergic diseases in 6–12-year-old children suffering from mild to moderate asthma [157]. A significant increase in serum IL-10 levels, and a reduction in asthma responses was observed in children administrated with β-glucans. These studies showed that β-glucans could be used for understanding, and treatment of, allergic and inflammatory diseases.

Juvonen et al. studied the effects of a modified oat bran beverage, containing β-glucans, on satiety-related gastrointestinal hormone responses in normal healthy male and female volunteers [158]. A greater postprandial increase in satiety, plasma glucose, cholecystokinin, insulin, and glucagons, such as peptide 1 and peptide YY, and a greater decrease in postprandial ghrelin were observed. The viscosity differences in oat bran beverage containing β-glucans strongly influenced short-term gut hormone responses and modulated postprandial satiety-related physiology. Carpenter et al. investigated the post-exercise immunosuppressive response of yeast-derived whole glucan particles (WGP) in male and female volunteers [159]. The supplementation of WGP-glucan significantly enhanced CD14^+^ and CD14^+^/CD16^+^, LPS-stimulated production of interferon-γ, and IL-2, IL-4, and IL-5. The study suggested that WGP-glucan has post-exercise immunoprotective abilities.

Lee et al. studied the immunomodulating effects of nutrients enriched with β-glucans in critically ill patients with pulmonary disease and trauma [160]. In a randomized, double-blind, placebo-controlled study, β-glucan-enriched fractions showed an increase in natural killer cell activities and serum albumin. However, a decrease in high sensitivity C-reactive protein was observed. In this study, nutrients enriched with β-glucans showed a beneficial effect on natural killer cell activity, which indicated that β-glucans can serve as an attractive candidate to stimulate protective immunity.

Talbott and Talbott investigated the effects of yeast β-glucan (Wellmure, WGP) supplement on mood state and upper-respiratory tract infection (URTI) symptoms in 18–53-year-old marathon runners [161]. The Talbott group later studied the effects of the same β-glucan (WGP) in 18–65-year-old moderate to highly-stressed men and women [162]. In another study, they reported the effects of β-glucans (WGP) on URTI symptoms and psychological well-being in women with moderate levels of psychological stress [163]. During these studies, a significant reduction in URTI symptoms, increased vigor, and overall health, as well as decreased fatigue, confusion, and tension were observed. These studies showed that a dietary supplement of Wellmure (WGP) could help to reduce upper respiratory tract infections and could maintain immune protection against daily stresses.

Lower and upper respiratory tract infections are common in children. β-Glucans have been proven as natural immunomodulators for the prevention and treatment of various respiratory-related disorders. Vetvicka et al. investigated the effects of yeast-derived β-glucans (glucan #300) in 8–12-year-old, male and female children with chronic respiratory problems [164]. A significant increase in changes in the production of CRP and lysozymes in children and a greater improvement in the general health condition of children was observed. Vetvicka and colleagues reported an increased production of salivary IgM, IgA, and IgG, as well as strongly-stimulated immunity in β-glucan (glucan #300)-fed children with chronic respiratory disorders [165]. Later, in another study, they reported a significant increase in exhaled nitric oxide and physical endurance from orally-administrated β-glucan in children with chronic respiratory disorders [166].

Richter et al. reported the effects of oral administration of yeast β-glucan in 8–12-year-old children with chronic respiratory disorders [167]. A significant increase in the production of CRP, lysozyme, and calprotectin was observed in children supplemented with β-glucan. In another study, Richter et al. reported a strong reduction of salivary cortisol and cotinine levels in yeast β-glucan-supplemented children [168]. A significant reduction in clinical problems of children affected with chronic respiratory disorders, and an increased physical endurance, was observed. Richter and colleagues later reported the effects of oral supplementation of yeast β-glucan on the physical activity and immune status of children with respiratory disorders [169]. A significant difference between male and female children in terms of physical endurance was observed. Additionally, a significant reduction in exhaled nitric oxide levels and a stabilization of salivary IgA levels were observed. These studies showed that short-term administration of yeast β-glucan (glucan #300) could stimulate mucosal immunity and regulate the energetic metabolism in children with chronic respiratory problems.

Jesenak et al. evaluated the effects of Imunoglukan P4H^®^ syrup on recurrent respiratory infections in children [170]. The administration of Imunoglukan P4H^®^ syrup in an open clinical trial showed a 50% reduction in the frequency of recurrent respiratory infections in children with no adverse effects. Jesenak and group, again, reported the effectiveness of Imunoglukan P4H^®^ syrup on the prevention of respiratory infections in children [171]. A double-blind, placebo-controlled, randomized, multicenter study showed the preventive effect of Imunoglukan P4H^®^ syrup in children with respiratory problems. A significant reduction in the frequency of flu and flu-like diseases, as well as respiratory tract infections, were observed. Imunoglukan P4H^®^ syrup significantly modulated cellular and humoral immunity. In another study, the Jesenak group reported the significant immunomodulating and anti-allergic effect of pleuran, isolated from *Pleurotus ostreatus* [172]. A significant reduction in peripheral blood eosinophilia and stabilized levels of total IgE in serum, in children with recurrent respiratory infections, were observed. Later, this same group investigated the immunomodulatory and anti-inflammatory activity of an Imunoglukan P4H^®^ cream, containing β-glucans (pleuran), in patients suffering from atopic dermatitis [173]. The topical application of Imunoglukan P4H^®^ showed significant improvements in both subjective and objective symptoms of atopic dermatitis and a significant decline in disease severity; exacerbation was observed. These studies suggest the potential of pleuran-containing creams and syrup (Imunoglukan P4H^®^) as a supportive complementary therapy for atopic dermatitis and respiratory disorders in children.

Grau et al. reported the immunomodulatory activity of β-glucans in children with respiratory tract infections [174]. A significant reduction in the average number of respiratory tract infection episodes and occurrence risk, as well as respiratory disorders, such as the common cold, otitis, laryngitis, pharyngitis, and bronchitis, were observed. Pasnik et al. also investigated the immunomodulatory effects of Imunoglukan P4H^®^ syrup, containing pleuran, on recurrent respiratory tract infections in children [175]. Similar to the above-mentioned studies, the Imunoglukan P4H^®^ syrup showed a significant reduction in the total number of respiratory tract infections during treatment. Additionally, a significant reduction in respiratory infections, such as otitis, flu, bronchitis, and laryngitis, was observed. Interesting, the syrup was well tolerated in children and no serious adverse effects were observed. These studies showed that β-glucans (pleuran) from *P. ostreatus* can be helpful in reducing respiratory tract infections in children with chronic respiratory disorders.

## 4. Bone Regeneration/Bone Injury Healing Effects of β-Glucans

The antiosteoporotic, bone healing, and bone regeneration effects of β-glucans have been extensively studied using in vitro, as well as animal- and human-based clinical trials. The antiosteoporotic effects of β-glucans are listed in Table 8, Table 9 and Table 10 and are described below.

### 4.1. Bone Regeneration/Bone Injury Healing Effects of β-Glucans—In Vitro Studies

The hematopoietic response of PGG-glucan on human bone marrow mononuclear cells was investigated by Turnbull et al. [176]. PGG-glucans significantly enhanced the bone marrow mononuclear myeloid colony formation factor. This study demonstrated that PGG-glucans can effectively enhance human hematopoietic activity on myeloid progenitors, independent of SCF, IL-3 or secondary cytokines.

Previously, we investigated the effects of a mixture of polycan and calcium lactate gluconate (polycalcium) on osteoporosis in murine osteoclast progenitor cells (RAW264.7) [177]. Osteoblast proliferation was stimulated, which prevented RANKL-induced osteoclast differentiation of RAW264.7 cells. In another study, the wound healing properties of β-glucans, isolated from *Aureobasidium pullulans*, were studied in human fetal dermal fibroblast cell lines [178]. β-Glucans mediated transforming growth factor (TGF-β1) showed increased procollagen production and a significantly-decreased optical density value at 570 nm. In addition, fibroblast proliferation and migration into wound defects were suppressed in a dose-dependent manner. These studies revealed the wound healing properties of β-glucans and suggested that it could be used as an anti-osteoporotic agent to accelerate bone formation and to inhibit bone resorption.

Przekora et al. studied the biomedical capabilities of chitosan/hydroxyapatite (chit/HA) and novel chitosan/bacterial β-glucan/hydroxyapatite (chit/glu/HA) materials as scaffolds for bone regeneration using human fetal osteoblast cell line [179]. The chit/HA scaffold showed better mechanical properties than chit/glu/HA; however, chit/glu/HA showed improved porosity, flexibility, and significantly higher water uptake capability. In addition, chit/glu/HA showed favorable osteoblast survival, proliferation and spreading. Except for poor mechanical properties, chit/glu/HA showed a better biomedical potential.

### 4.2. Bone Regeneration/Bone Injury Healing Effects of β-Glucans—Animal Studies

The inhibitory response of barley β-glucans on chromosomal aberrations in bone marrow and spermatogonial cells of CD1 mice was investigated by Tohamy et al. [180]. A significant reduction in a total number of cells with structural chromosomal aberrations was observed. This study showed that barley β-glucans have a significant role in reducing genotoxicity induced by anti-neoplastic drugs during cancer chemotherapy. Previously, we determined the optimum composition of polycan and calcium lactate-gluconate complex with optimum activity in ovariectomy-induced osteoporotic rats [181]. A significant decrease in ovariectomy-induced osteoporotic changes was observed at a ratio of 10:90. The continuous administration of polycal preserved bone mass and strength. In another study, we investigated the synergistic effects of polycalcium (polycan + calcium lactate gluconate) in an osteoarthritis rat model [182]. Osteoarthritis-related changes in rats were inhibited after continuous administration of polycal for 28 consecutive days, and anti-osteoarthritis effects, including induction of chondrocyte proliferation, were observed.

Park et al. investigated the inhibitory activities of polycal (polycan + calcium lactate gluconate) on ligation induced-experimental periodontitis and related alveolar bone loss [183]. The continuous topical application of polycal significantly inhibited bacterial proliferation, periodontitis, and alveolar bone loss. These studies showed the beneficial synergistic effects of polycan and calcium lactate gluconate formulations against osteoarthritis. Borkowski et al. investigated the synergistic effects of carbonated hydroxyapatite (CHAP) granules and β-glucans as a filler for bone defects in New Zealand white rabbits [184]. Significant integration of implants with bone tissue, as well as no signs of graft rejection, were observed, and the biomaterial showed a stimulating effect on bone formation and mineralization. This study highlighted the potential application of β-glucans in bone tissue regeneration and as a filler in bone defects.

Ku et al. evaluated the effects of polycan isolated from *Aureobasidium pullulans* in different osteoporosis model rats [76]. A significant increase in bone mineral density of the femur, tibia, and L6, as well as an increase in calcium bioavailability and a decrease in calcium secretion in ovariectomy thyroparathyroidectomy rat models, were observed. This study showed that calcium bioavailability improved the properties of polycan in bones. Jung et al. also investigated the protective effects of polycan isolated from *A. pullulans* in ovariectomy-induced osteoporosis [77]. Polycan treatment inhibited ovariectomy-induced alterations in bone resorption and turnover in a dose-dependent manner; however, a significant increase in histomorphometrical indices of bone formation and serum expression levels of bALP were observed. Polycan preserved bone mass and strength and increased the rate of bone formation, supporting the idea that polycan can be used as an anti-osteoporosis agent.

### 4.3. Bone Regeneration/Bone Injury Healing Effects of β-Glucans—Clinical Studies

Previously, we reported on the significant antiosteoporotic effects of polycalcium in in vitro and animal-based studies [177,178,181,182]. We also estimated the antiosteoporotic effects of polycalcium in human-based clinical trials [185]. An open-label, single-center trial of polycalcium in 40–60-year-old healthy women demonstrated significant changes in urinary deoxypyridinoline levels, bone-specific alkaline phosphatase, serum osteocalcin, urinary cross-linked N-telopeptide of type-1 collagen, urinary cross-linked C-telopeptide of type-1 collagen, calcium, and phosphorus levels. Additionally, polycalcium, at a dose of 400 mg, showed significant efficiency for improving bone metabolism and was well tolerated.

The efficacy and safety of polycan on bone biochemical markers in healthy perimenopausal women were studied by Kim et al. [186]. A randomized, double-blind, placebo-controlled study showed significant changes in osteocalcium, deoxypyridinoline, and bone-specific alkaline phosphatase levels, and no significant difference in adverse event during the safety assessment test were observed. However, after four weeks of polycan treatment, no statistically-significant results on bone metabolism biomarkers were observed. Further studies in a large population and a longer treatment period are needed to confirm the findings.

## 5. Anti-Diabetic/Anti-Obesity Effects of β-Glucans

The anti-diabetic and anti-obesity effects of β-glucans have been largely studied in animal- and human-based clinical trials, but have not been reported using in vitro studies. The anti-diabetic and anti-obesity effects of β-glucans are listed in Table 11 and Table 12, and are described below.

### 5.1. Anti-Diabetic/Anti-Obesity Effect of β-Glucans—In Vitro Studies

To the best of our knowledge, there are no available reports on in vitro studies of the anti-diabetic and/or anti-obesity effects of β-glucans.

### 5.2. Anti-Diabetic/Anti-Obesity Effect of β-Glucans—Animal Studies

The effects of lentinan from *L. edodes* and a polysaccharide glucan from *A. blazei* on cytochrome P450s (CYPs) expression in female BALB/c mice were investigated by Hashimoto et al. [187]. Lentinan and *A. blazei-*derived polysaccharide glucans both suppressed constitutive and 3-methylcholanthrene-induced CYP1A expression and ethoxyresorufin-O-deethylation activity in the liver. Neyrinck et al. studied the efficiency of fungal-derived chitin-glucan in modulating gut microbiota, as well as glucose and lipid metabolism, in high-fat-diet induced obese mice [188]. Significant cecal enlargement, with prominent changes in gut microbiota, was observed. In addition, chitin-glucan significantly decreased body weight gains, fat mass development, glucose intolerance, fasting hyperglycemia, hepatic triglyceride accumulation, and hypercholesterolemia. The chronic consumption of chitin-glucan positively affected the development of obesity and associated metabolic disorders by restoring the composition and/or activity of gut microbiota.

Lim et al. studied the anti-diabetic effects of polycan isolated from *A. pullulans* on high-fat-diet (HFD)-induced hyperlipemia and hepatic damage in hamsters [75]. Polycan did not cause a significant change in body weight and food consumption by hamsters. However, a significant reduction in serum levels of triglyceride, alanine aminotransferase, aspartate aminotransferase, total-, and LDL-cholesterol, as well as levels of arteriosclerosis, were observed. This study showed the favorable effects of polycan in protecting from liver damage, as well as in reducing HFD-induced hyperlipemia and associated arteriosclerosis. Sohn et al. examined the combined effects of β-glucan isolated from *A. pullulans* and *Folium mori* extract (BG-FM) for treating diabetes, complicated hepatopathy, and nephropathy using streptozotocin-induced diabetic rats [189]. The BG-FM formulations showed a significant reduction in hyperglycemia, hepatopathy, and nephropathy of body weight changes. The synergistic effects of the BG-FM complex at a 1:4 ratio was most effective in suppressing hyperglycemia and diabetic response.

### 5.3. Anti-Diabetic/Anti-Obesity Effect of β-Glucans—Clinical Studies

Tappy et al. investigated the glycemic and insulinemic response of breakfast cereal, containing oat-derived β-glucans, in 49–57-year-old men and women suffering from non-insulin-dependent diabetes mellitus [190]. Oat β-glucan showed a dose-dependent increase in plasma glucose. Bourdon et al. investigated postprandial glycemic response of barley-derived β-glucan-containing meals in healthy men [191]. Plasma insulin, glucose, cholecystokinin, and triacylglycerol concentrations increased significantly in all test meals, but barley-containing meals showed a higher insulin response. Cavallero et al. also investigated the effects of barley-derived β-glucan on glycemic response in 20–27-year-old healthy, non-diabetic men and women [192]. A linear decrease in glycemic response and postprandial blood glucose levels were observed. The barley-containing meals appeared to stimulate reverse cholesterol transport, which may contribute to the cholesterol-lowering ability of barley.

Jenkins et al. investigated the effects of oat-derived β-glucans on glycemic response in 59–63-year-old men and women suffering from type 2 diabetes [193]. A randomized, open-labeled, crossover study with type 2 diabetic patients showed a significant reduction in glycemic index, but maintained palatability of the test food. Tapola et al. also investigated the postprandial glycemic response of oat bran containing β-glucans in 61–73-year-old patients suffering from type 2 diabetes [194]. In a randomized, controlled, repeated measure design, oat bran, high in β-glucans, showed a reduction in glycemic response, and actively decreased postprandial glycemic response in type 2 diabetic patients.

Biorklund et al. investigated the postprandial glycemic responses of beverages enriched with oat- or barley-derived β-glucans [195]. A single blind, controlled study was conducted in parallel groups of 18–70-year-old healthy men and women, with mildly elevated cholesterol concentrations. The oat-derived β-glucans significantly lowered total cholesterol, postprandial glucose, and insulin concentrations; whereas barley-derived β-glucans did not show significant responses. Mäkeläinen et al. investigated the physiological responses and the effects of oat-derived β-glucans on insulin and glycemic indeces in male and female volunteers [196]. Significant reductions in insulin and glycemic indeces were observed with the administration of oat β-glucans. Granfeldt et al. investigated the postprandial insulinemia and glycemic responses of a muesli product containing oat β-glucans [197]. The muesli product, containing 4 g of oat-derived β-glucans, significantly reduced insulin and glucose responses in healthy people.

Thonder and Henry investigated the glycemic response of food products (Chapati) fortified with barley β-glucans [198]. A randomized, single-blind, controlled, crossover study was conducted in 26–50-year-old healthy men and women. The study showed a significant reduction in glucose, and postprandial blood glucose concentrations, as well as glycemic index. Cugnet-Anceau et al. investigated the effects of diet formulations enriched with oat-derived β-glucans in 30–75-year-old type 2 diabetic men and women [199]. A parallel, randomized, double-blind, placebo-controlled study showed a significant reduction in triacylglycerides while no significant differences in fasting glucose, HbAa1c, apoB, and total-, LDL-, and HDL- cholesterol were observed. In this study, a low-dose of β-glucans did not improve the metabolic profile of type 2 diabetic patients.

## 6. Cholesterol and Blood Pressure Lowering Effects of β-Glucans

The cholesterol and/or blood pressure lowering effects of β-glucans have been largely studied in human-based clinical trials, but are rarely reported using in vitro or animal-based in vivo studies. The cholesterol and blood pressure lowering effects of β-glucans are listed in Table 13 and Table 14, and are described below.

### 6.1. Cholesterol and Blood Pressure Lowering Effects of β-Glucans—In Vitro Studies

To the best of our knowledge, there are no available reports of in vitro studies on the cholesterol and/or blood pressure lowering effect of β-glucans.

### 6.2. Cholesterol and Blood Pressure Lowering Effects of β-Glucans—Animal Studies

Vetvicka and Vetvickova investigated the effects of yeast-derived β-glucans on blood cholesterol levels and macrophage functionality in mice [200]. A significant dose-dependent decrease in plasma cholesterol and triglycerides was observed. Kusmiati and Dhewantata investigated the anti-cholesterolemic effects of yeast-derived β-glucans in Sprague-Dawley rats [201]. Yeast-derived β-glucans significantly reduced the total cholesterol in blood plasma and liver to a normal level. Additionally, a significant decrease in triglyceride and malondialdehyde levels were observed.

### 6.3. Cholesterol and Blood Pressure Lowering Effects of β-Glucans—Human Studies

The hypocholesterolemic response of oat-derived β-glucans in 30–65-year-old men and women with elevated LDL-cholesterol levels was investigated by Davidson et al. [202]. A significant dose-dependent reduction in LDL-cholesterol levels was observed, which supports the idea of independent hypochloesterolemic effect of oat-derived β-glucans. Bourdon investigated the cholesterol-lowering response of barley β-glucans in healthy men [191]. β-glucan-containing meals showed a significant increase in plasma insulin, glucose, cholecystokinin, and triacylglycerol levels. The consumption of meals enriched with barley β-glucans appeared to stimulate reverse cholesterol transport, which showed the cholesterol-lowering ability of barley β-glucans. Nicolosi et al. studied the cholesterol-lowering effects of yeast-derived β-glucans in hypercholesterolemic, obese men [203]. Yeast-derived β-glucan consumption significantly reduced plasma total-, LDL-, and HDL-cholesterol concentrations; however, changes in triacylglycerol concentrations were non-significant.

Lovergrove studied the effects of low doses of oat bran, containing β-glucans in mild- to moderate-hyperlipidemic volunteers [204]. A randomized, double-blind, parallel study was conducted in 30–70-year-old men and women with slightly-elevated LDL-cholesterol levels. A significant decline in HDL-cholesterol concentrations was observed. However, the low-dosage of β-glucans (3 g/day) showed no significant reduction in plasma total cholesterol, LDL-cholesterol, insulin, and glucose concentrations, which shows that oat β-glucans, at a low dose, are not sufficient to lower cholesterol. Jenkins investigated the effects of oat-derived β-glucans in reducing serum lipid risk factors for cardiovascular disease in hyperlipidemic adults [205]. A significant reduction in total-, total:HDL-, LDL:HDL-cholesterol and apolipoprotein B:A-I, as well as a reduction in cardiovascular disease risk, were observed in the high-fiber fed group.

Keogh et al. investigated the effects of barley-derived β-glucan formulations for reducing cardiovascular disease risk factors in 18–65-year-old, mildly-hyperlipidemic men [206]. No significant changes in total-, LDL-, and HDL-cholesterol, fasting glucose, triacylglycerol or postprandial glucose were observed. Kerckhoffs et al. investigated the effects of oat-derived β-glucans on serum lipoproteins in 18–65-year-old, mildly-hypercholesterolemic men and women [207]. A minor decrease in LDL-cholesterol and a total:HDL-cholesterol ratio was observed in a β-glucan-rich drink-fed group. However, triacylglycerol and HDL-concentrations showed non-significant differences.

Behall et al. investigated the cardiovascular disease risk factor reducing activity of barley-derived β-glucans in 28–62-year-old, moderately-hypercholesterolemic men [25]. Significant reductions in total- and LDL-cholesterol, and triacylglycerol concentrations, were observed. However, HDL-cholesterol concentrations increased. Behall and colleagues, later, compared different sources of soluble dietary fiber, including barley-derived β-glucans, and their effect in reducing cardiovascular disease risk factors in 38–53-year-old, mildly-hypercholesterolemic men and women [26]. A significant reduction in total cholesterol, as well as LDL particle size, was observed in diets containing barley-derived β-glucans; HDL-cholesterol and triacylglycerol concentrations showed non-significant differences. These studies suggested that cardiovascular disease risk factors might be reduced by consuming foods rich in higher concentrations of soluble fiber.

He et al. investigated the blood pressure reducing effects of oat dietary fiber (β-glucans) [208]. A randomized, double-blind, placebo-controlled study was conducted on 30–65-year-old volunteers with elevated blood pressure or stage-1 hypertension. Maki et al. studied the effects of foods rich in oat-derived β-glucans on carbohydrate homeostasis, blood pressure, and oxidative stress [209]. A randomized, double-blind, controlled study was conducted on >40-year-old men and women with elevated blood pressure. Significant changes in insulin were observed while blood pressure response and biomarkers of oxidative stress showed non-significant differences. The oat-dietary fiber intake showed a positive effect in lowering systolic as well as diastolic blood pressure, suggesting that oat-derived β-glucans-rich dietary fibers could help to lower blood pressure.

Queenan et al. studied the physiological effects of oat-derived β-glucans on cardiovascular disease in 22–65-year-old hypercholesterolemic men and women [109]. Oat-derived β-glucan concentrate consumption (6 g/day) significantly reduced total- and LDL-cholesterol concentrations. Shimizu et al. investigated the effects of rice diet substitution with barley-derived β-glucans on the reduction of cholesterol and visceral fat areas in 30–60-year-old hypercholesterolemic Japanese men [210]. A randomized, double-blind, placebo-controlled study showed a significant reduction in serum total- and LDL-cholesterol concentrations, as well in visceral fat areas. Liatis et al. investigated the effects of oat-derived β-glucan-enriched bread consumption on glucose homeostasis and lipid profiles in 50–70-year-old patients with type 2 diabetes [27]. A randomized, double-blind study showed a significant reduction in total- and LDL-cholesterol, insulin resistance, and fasting plasma insulin levels. The consumption of oat-derived β-glucans-enriched bread showed improvements in insulin resistance and lipid profiles in patients with type 2 diabetes.

## 7. Antigenotoxic/Antimutagenic/Antioxidative Effects of β-Glucans

The antigenotoxic, antimutagenic, and/or antioxidative effects of β-glucans have been largely studied using in vitro and animal-based in vivo studies, but are rarely reported in human-based clinical trials. The antigenotoxic, antimutagenic or antioxidative effects of β-glucans are listed in Table 15 and Table 16, and are described below.

### 7.1. Antigenotoxic/Antimutagenic/Antioxidative Effects of β-Glucans—In Vitro Studies

The antigenotoxic effects of yeast-derived β-glucans and fungus-derived β-glucan-chitin complex in V79 hamster lung cells were investigated by Slamenova et al. [211]. The comet assay exhibited a significant reduction in genotoxicity, indicating the protective effect of the tested β-glucans against oxidative damage to DNA caused by scavenging of singlet oxygen or OH radicals. Krizkova et al. investigated the antigenotoxic and antioxidant activity of yeast-derived mannan and mannan conjugates with microbial enzyme penicillin G acylase and human serum albumin in the unicellular flagellate, *Euglena gracilis* [212]. The tested β-glucan formulations showed significant protective antigenotoxic and antioxidative activities against acridine orange and ofloxacin. Oliveira et al. investigated the antimutagenic activity of barley-derived β-glucans, using a micronucleus assay in HTC and CHO-k1 cell lines [213]. β-Glucans showed a significant chemoprotective activity against methylmethane sulfonate-induced DNA damage in CHO-k1 cells. The study showed that barley-derived β-glucans have bioantimutagenic and desmutagenic activities.

Angeli et al. investigated the genotoxic and antigenotoxic effects of β-glucans isolated from *A. brazei* Murrill in human peripheral lymphocytes [214]. A significant dose-dependent protective effect against DNA damage induced by H_2_O_2_ and trp-P-2 was observed. Later, Angeli and colleagues investigated the clastogenic and anti-clastogenic effects of barley-derived β-glucans in CHO-k1 and HTC cells [215]. The barley-derived β-glucans showed a significant anti-clastogenic effect against methylmethane sulfonate and 2-aminoanthracene induced DNA damage in the tested cell lines. In another study, Angeli’s group investigated the chemoprotective effects of β-glucans isolated from *A. blazei* in human hepatoma cell lines [216]. The mushroom (*A. blazei*)-derived β-glucan did not show mutagenic or genotoxic effects, but showed a chemoprotective effect against DNA damage caused by B[a]P. This study suggested that the mushroom-derived β-glucan might modulate cell metabolism. Xia et al. investigated the antioxidative activities of chrysolaminarin from *Odontella aurita* [101]. Chrysolaminarin exhibited a significant hydroxyl radical scavenging activity, but was less effective in reducing the 1-diphenyl-2-picrylhydrazyl (DPPH)-radical scavenging activity.

### 7.2. Antigenotoxic/Antimutagenic/Antioxidative Effects of β-Glucans—Animal Studies

Yamamoto et al. investigated the antimetastatic and antiangiogenic effects of mushroom-derived β-glucans (SBG), extracted from *Sparassis crispa*, in ICR and C57BL/6J mice [74]. SBG suppressed B16-F10 cell-induced angiogenesis in female ICR mice; in addition, vascular endothelial growth factor induced neovascularization in female C57BL/6J mice. In addition, it suppressed the growth and number of metastatic tumor foci in the lungs of female C57BL/6J mice. This study showed the suppressive effect of SBG against tumor growth and metastasis in lung, as well as the inhibition of tumor-induced angiogenesis. Erkol et al. investigated the effects of yeast-derived β-glucans on oxidative damage to liver during obstructive jaundice in Wistar albino rats [217]. A significant reduction in levels of alanine and aspartate aminotransferases, lactate dehydrogenase, gamma-glutamyl transpeptidase in serum, and levels of lipid peroxide and malondialdehyde in the liver were observed. However, significantly greater glutathione and superoxide dismutase levels were observed in groups administrated yeast-derived β-glucans. This study showed the phagocytotic and antioxidative activities of yeast-derived β-glucans in reducing liver damage and oxidative stress in obstructive jaundice.

For the last decade, the increasing trend of using irradiation-emitting devices has been observed in medical, industrial, domestic, and military applications. Ceyhan et al. investigated the effects of electromagnetic radiations on the antioxidant status of skin, and the possible protective effects of yeast-derived β-glucans against oxidative injury in Wistar albino rats [218]. β-Glucans significantly reversed the elevation of malondialdehyde levels and superoxide dismutase activity caused by irradiation exposure. In addition, yeast-derived β-glucans slightly enhanced catalase activity and prevented the depletion of glutathione peroxidase activity, caused by electromagnetic radiation. Pillai and Devi investigated the radioprotective effect of mushroom β-glucans, extracted from *Ganoderma lucidum*, in young swiss albino mice [219]. A significant increase in post-irradiation mouse survival, and a reduction in the number of aberrant cells was observed. These studies demonstrated the antioxidative, and radioprotective activities of yeast-derived β-glucans, through which β-glucans could ameliorate the oxidative skin injury caused by electromagnetic radiation.

### 7.3. Antigenotoxic/Antimutagenic/Antioxidative Effects of β-Glucans—Clinical Studies

To the best of our knowledge, there are no reports available on clinical studies of antigenotoxic/antimutagenic, and/or antioxidative effects of β-glucans.

## 8. Conclusions and Future Perspectives

The use of fibers and polysaccharides from mushrooms, yeasts, and cereals have been widely documented, along with their observed antitumor, anti-microbial, anti-allergic, as well as immune-modulating effects, in addition to the cardiovascular disease risk-reducing activities, commonly attributed to their bioactive compound, β-glucans. β-Glucans are natural bioactive compounds and can be taken orally as a food supplement or as part of a daily diet and are considered safe to use. When polysaccharide glucans are included in a meal, the rate of carbohydrate and lipid absorption slows down, ultimately modifying the alimentary hormone and lipid responses. The crude extracts or purified β-glucans have been clinically used as part of therapy for cancer and other infectious diseases. In 1997, the US Food and Drug Administration registered oat bran (3 g β-glucan/day dosage) as the first cholesterol-reducing food. Furthermore, β-glucans mobilized murine progenitor cells from bone marrow as well as enhanced murine hematopoietic recovery following bone marrow injury.

β-Glucans possess strong immune-modulatory and anti-osteoporotic activities, which have been proved by in vitro and animal- and human-based clinical trials. The anti-diabetic and/or anti-obesity effects of β-glucans have been largely studied in animal- and human-based clinical trials, but have not been reported in in vitro studies. The cholesterol and/or blood pressure lowering effects of β-glucans have been largely studied in human-based clinical trials, but are rarely reported through in vitro or animal-based in vivo studies. The antitumor, antigenotoxic, antimutagenic and/or antioxidative effects of β-glucans have been widely studied using in vitro and animal-based in vivo studies, but human-based clinical trials are rarely reported. The medical significance and effectiveness of β-glucans, as antimicrobial, anticancer, anti-diabetic and anti-hyperchloresterolemic polysaccharides, have been reviewed [20,29,38]. However, systematic study of the clinical and physiological significance of β-glucans is scarce. In this review, we discussed the medical significance of β-glucans through in vitro, as well as through animal- and human-based clinical studies.

## Figures and Tables

**Table 1:** Common bioactive β-glucans, their structure, and sources.

β-Glucan	Abbreviation	Source	Structure	Reference
**Fungal β-Glucan**				
Schizophyllan/Sizofiran/Sonifilan	SPG	*Scizophyllum commune*	Linear (1,3) β-glucan with (1,6)-linked-β-glucosyl or β-oligoglucosyl side chain	[39,40]
Sclerotinan/Sclerotan	SSG	*Sclerotinia sclerotiorum*, *Sparassis crispus*	Linear (1,3) β-glucan with (1,6)-linked-β-glucosyl or β-oligoglucosyl side chain	[39,41,42]
Scleroglucan/Sclero-β-glucan	SR-glucan	*Sclerotium rolfsii*, *Sclerotium glucanicum*	Linear (1,3;1,6) β-glucan	[43,44]
Pestalotan	-	*Pestalotia* sp.	Linear (1,3) β-glucan with (1,6)-linked-β-glucosyl or β-oligoglucosyl side chain	[45]
Epiglucan	-	*Epicoccum nigrum*	Linear (1,3) β-glucan with (1,6)-linked-β-glucosyl or β-oligoglucosyl side chain	[46]
Pachymaran/Pachyman	-	*Poria cocos*	Linear (1,3) β-glucan	[47,48]
T-4-N, T-5-N	-	*Dictyophora indusiata* Fisch, *Phallus indusiata*	Branched (1,3;1,6) β-glucan	[49]
β-glucan	-	*Glomerella cingulata*	Branched (1,3;1,6) β-glucan	[50,51]
Grifolan	GRN	*Grifola frondosa*	Linear (1,3) β-glucan with (1,6)-linked-β-glucosyl or β-oligoglucosyl side chain	[52,53,54]
Lentinan	LNT	*Lentinula edodes*	Linear (1,3) β-glucan with (1,6)-linked-β-glucosyl or β-oligoglucosyl side chain	[55,56,57]
LC11	-	*Lentinus edodes*	Branched (1,3;1,4) β-glucan	[57]
Coriolan	-	*Coriolus versicolor*	Linear (1,3) β-glucan with (1,6)-linked-β-glucosyl or β-oligoglucosyl side chain	[58]
Krestin	PSK	*Trametes versicolor*	Protein-bound linear (1,3) β-glucan	[59]
Pleuran	HA-glucan	*Pleurotus tuber-*regium, *Pleurotus ostreatus*	Branched (1,3;1,6) β-glucan	[60]
β-glucan	MFL-glucan	*Monilinia fructicola*	Branched (1,3;1,6) β-glucan	[61]
β-glucan	MFN-glucan	*Monilinia fructigena*	Branched (1,3;1,6) β-glucan	[61]
β-glucan	AM-ASN	*Amanita muscaria*	Branched (1,3;1,6) β-glucan	[61]
β-glucan	AAG	*Auricularia auricular*-judae	Branched (1,3;1,6) β-glucan	[62]
Tylopilan	-	*Tylopilus felleus*	Branched (1,3;1,6) β-glucan	[63,64]
β-glucan	-	*Cryptoporus volvatus*	Branched (1,3;1,6) β-glucan	[65]
β-glucan	-	*Pythium aphanidermatum*	Branched (1,3;1,6) β-glucan	[66]
Polysaccharide-glucan	PS-G	*Ganoderma lucidum*	Branched (1,3;1,6) β-glucan	[67,68]
β-glucan	-	*Agaricus blazei*	Branched (1,3;1,6) β-glucan	[69,70]
β-glucan	-	*Cordyceps sinensis*	Branched (1,3;1,6) β-glucan	[71,72]
β-glucan	HEP3	*Hericium erinaceus*	Branched (1,3;1,6) β-glucan	[73]
β-glucan	SBG	Sparassis crispa	Branched (1,3;1,6) β-glucan	[74]
Polycan	-	*Aureobasidium pullulans*	Branched (1,3;1,6) β-glucan	[75,76,77]
β-glucan	BG-PN	*Pholiota nameko*	Branched (1,3;1,6) β-glucan	[78]
Pendulan	-	*Porodisulus pendulus*	Linear (1,3) β-glucan with (1,6)-linked-β-glucosyl or β-oligoglucosyl side chain	[79]
**Lichen β-Glucan**				
Pustulan	-	*Gyrophera esculenta*, *Umbiliaria papulosa*	Linear (1,3) β-glucan	[80]
Lichenan/Lichenin	-	*Cetraria islandica*	Linear (1,3;1,4) β-glucan	[80]
**Yeast β-Glucan**				
Zymosan	-	*Saccharomyces cerevisiae*	Branched (1,3;1,6) β-glucan	[81,82,83]
Βetafectin/TH-glucan	PGG	*Saccharomyces cerevisiae*	Branched (1,3;1,6) β-glucan	[84,85]
Yeast whole β-glucan particles	WPG, WGPs	*Saccharomyces cerevisiae*	Yeast whole β-glucan particles	[86,87]
β-glucan	MG	*Saccharomyces cerevisiae*	Linear (1,3) β-glucan	[81]
β-glucan	IS-2	*S. cerevisiae* (Mutated)	-	[88]
Yestimun	-	*Saccharomyces cerevisiae*	Branched (1,3;1,6) β-glucan	[89]
Cerevan	-	*Saccharomyces cerevisiae*	Branched (1,3;1,6) β-glucan	[90]
**Bacterial β-Glucan**				
Curdlan	-	*Alcaligenes faecalis*, *Agrobacterium rhizogenes*, *Agrobacterium radiobacter*	Linear (1,3) β-glucan	[91,92,93,94]
β-glucan	DMJ-E	*Agrobacterium* sp. *R259*	Linear (1,3) β-glucan	[95]
**Seaweed/Algal β-Glucan**				
Laminaran/Laminarin	-	*Laminaria* sp. (brown algae), *Laminaria cichorioides*	Linear (1,3) β-glucan with (1,6)-linked-β-glucosyl or β-oligoglucosyl side chain	[96,97]
Mycolaminarin	-	*Phytophthora* sp.	Linear (1,3) β-glucan with (1,6)-linked-β-glucosyl or β-oligoglucosyl side chain	[98,99]
Chrysolaminarin	CL-2	*Ochromonas malhamensis*, *Odontella aurita*, *Chaetoceros muelleri*	Linear (1,3) β-glucan with (1,6)-linked-β-glucosyl or β-oligoglucosyl side chain	[100,101,102]
Phycarine	-	*Laminaria digitata*	Linear (1,3) β-glucan	[103]
Paramylon	-	*Euglena gracilis*, *Pavlova mesolychnon*	Linear (1,3) β-glucan	[104,105]
Leucosin	-	*Phaeodactylum tricornutum*	Linear (1,3) β-glucan with (1,6)-linked-β-glucosyl or β-oligoglucosyl side chain	[106]
**Cereal β-Glucan**				
Barley β-glucan	-	*Hordeum vulgare* L.	Linear (1,3;1,4) β-glucan	[107,108]
Oat β-glucan	-	*Avena sativa* L.	Linear (1,3;1,4) β-glucan	[109,110]
Wheat β-glucan	-	*Triticum vulgare*	Linear (1,3;1,4) β-glucan	[111,112]

**Table 2:** Antitumor effects of β-glucans—in vitro study.

β-Glucan	Cell Line	Analysis	Results	Reference
Fungal β-glucan	Human PBMC cell line	Cytokine inducing activity, TNF-α activity	Increased TNF-α activity.	[113]
Barley β-glucan	CHO-k1 cell line, and HTC cell line from *Ratus novergicus*	Micronucleus test in bi-nucleated cells to check mutagenicity	Chemoprotective and antimutagenic activity.	[114]
Polysaccharide-glucan from different sources	Human dendritic cells	Cell proliferation assay, FITC-dextran endocytosis assay, and ELISA	*Ganoderma lucidum* isolated polysaccharide significantly induced human PBMC proliferation and production of IL-10, and IL-12.	[115]
Yeast p-β-glucan (WGP, PGG)	BMDC, CD4^+^ T cells, MUC1-trasfected lymphoma RMA cells, Ovalbumin-transfected mammary adenocarcinoma cell line	T-cell differentiation assay, and Fluorescence-based neutrophil-mediated in vitro killing assay	Activated DCs and macrophages, promoted Th1 and cytotoxic T-lymphocyte priming and differentiation.	[87]
Mutated yeast β-glucan	Highly metastatic cell line of colon 26 carcinoma, colon 26-M3.1 and B16-BL6 melanoma cells, L5178Y-ML25 lymphoma cells, and mouse splenocytes	Antitumor and immunostimulating activities, Cytotoxicity analyses, and NK cell activity	Enhanced splenocyte proliferation activity in a dose-dependent manner, Increased NK cytotoxicity against Yac-1 tumor cells but did not affect the growth of colon 26-M3.1 cells.	[88]
Curdlan	Mo-DCs from healthy human volunteers and Leukemic cell line (THP-1)	ELISA, and RT-PCR	Th17-inducing activity.	[116]
Oat low molecular weight β-glucan (1,3;1,4)-β-d-glucan	Human Me45 cell line, Mouse macrophage cell line (P388/D1), Human HaCaT cell line, Human carcinoma A431 cell line	MTT assay, Cloning efficiency test, and Caspase-12 expression assay	Decreased cell viability of cancer cells while no toxicity to normal cells.	[117]
Fungal β-glucan	Sarcoma-180 cell line	Limulus amebocyte lysate coagulation test, Binding of Congo red, Toxicity test by brine shrimp assay, and MTT assay	Not toxic to brine shrimp assay.	[118]
Yeast β-glucan (WGP), Soluble β-glucan (NSG), Barley β-glucan	Lewis lung carcinoma cell line transfected with human MUCI (LL/2-MUCI), and Murine macrophage cell line J774	Analysis of macrophage degradation, and Analysis of bioactivity	Enhanced tumor regression and antitumor activity.	[119]
Lentinan	Sarcoma 180 tumor cell line	SEC-LLS measurements, Viscometric analysis, and MTT assay	Maximum inhibition ratio against Sarcoma-180 tumor cell growth.	[120]
Phycarine, Lentinan	BALB/c mouse-derived mammary tumor cell line Ptas64, Murine tumor cell line Yac-1, Blood from healthy volunteers	Flow cytometry, Phagocytosis, and Cytokine evaluation	Increased NK cell-mediated killing of tumor cell.	[121]

PBMC: Peripheral blood mononuclear cells; TNF-α: Tumor necrosis factor-α; CHO-k1: Chinese hamster ovarian cell line; HTC: Hepatoma cell lines; NK cells: Natural killer cells; FITC: Fluorescein isothiocyanate; ELISA: Enzyme-linked immunosorbent assay; IL: InterLeukin; BMDC: Bone marrow-derived dendritic cell; CD: Cluster of differentiation; MUC1: Mucine-1; Lymphoma RMA cell: Rauscher’s virus-induced lymphoma cell; Th: T-lymphocyte; DC: Dendritic cell; Mo-DCs: Monocyte-derived dendritic cells; RT-PCR: Reverse transcription-Polymerase chain reaction; Me45: Human pigmented malignant melanoma; HaCaT: Human normal keratinocytes; MTT assay: Mitochondrial metabolic function assay; SEC-LLS: Size-exclusion chromatography combined with multiangle laser light scattering.

**Table 3:** Antitumor effects of β-glucans—animal study.

β-Glucan	Organism	Analysis	Results	Reference
Fungal β-glucan (OL-2)	Specific pathogen-free male ICR mice	Physiochemical properties, NMR, Congo-red assay, and Antitumor activity assay	Low or no antitumor activity against solid form of Sarcoma-180. However, significant antitumor activity against ascites form of Sarcoma-180 and MH-134.	[122]
Fungal β-glucan (OL-2-I, II, III)	Male ICR mice	GLC, GLC-MS, and Antitumor activity assay	Antitumor activity against Sarcoma-180 tumor.	[123]
Fungal β-glucan (H-3-B; S-H-3-B)	ICR-JCL female mice	Electron microscopy, NMR spectroscopy, and Antitumor activity assay	Antitumor activity against Sarcoma-180 tumor.	[65]
Fungal β-(1,3)-glucan	Male ICR albino mice, transfected with Sarcoma-180 tumor cells	VDH response, and Mitogenic test	Triggered proliferation of splenic lymphocytes, vascular dilation, and VDH response.	[117]
Commercial Sonifilan	Male ICR mice	NMR, MALDI-MS, VDH reaction, and Congo Red test	Antitumor activity against solid Sarcoma-180 tumor, strong vascular dilation, and hemorrhage reaction. Enhanced hematopoietic response to cyclophosphamide induced leukopenic mice.	[124]
Grifolan LE (GRN), Commercial Sonifilan	5-weeks old male ICR mice	Antitumor activity assay, NMR, and ELISA	Antitumor activity against the solid form of Sarcoma-180 tumor.	[125]
Fungal β-glucan	BALB/c mice	Adoptive transfer test, Chemotactic factor assay, and Antitumor activity assay	Significant macrophage chemotactic factor activity. Increased IAP levels in serum, and inhibited growth of Meth-A tumor.	[126]
Yeast β-glucan (WGP), Soluble β-glucan (NSG), Barley β-glucan	Normal C57BL/6 mice deficient in either C3 or CR3 and their wild-type littermates	Analysis of elicited peritoneal granulocytes, peritoneal granulocyte-mediated, and splenic macrophage-mediated cytotoxicity	Barley and yeast β-glucans showed enhanced tumor regression and survival, and killed iC3b-opsonized tumor cells in bone marrow.	[119]
Wellmune + anti-tumor mAb therapy	6-weeks old male C57/B16 mice, transfected with human MUC1 lymphoma, in combination with mAb	Measurement of cytokine secretion in murine peritoneal macrophages, and BMDCs	Increased production of cytokine IL-2 in DCs.	[127]
Lentinan	BALB/c and C3H He/N and C3H He/J	Determination of EROD activity, and CYP1As levels, and DNA-binding activities of NF-κB and AhR	Suppression of CYP1As, decrease in EROD and DNA-binding activity of AhR, and decreased production of TNF-α.	[128]
Lentinan (L-FV-IB)	8-weeks old male BALB/c mice	Tumor weights, inhibition ratio, and enhancement ratio of body weight	Maximum inhibition ratio against Sarcoma-180 solid tumor.	[120]
Phycarine, Lentinan	6–10 week old female BALB/c mice	Flow cytometry, Phagocytosis, and Cytokine evaluation	Significantly stimulated phagocytic activity.	[121]
Yeast p-β-glucan (WGP, PGG)	Wild type C57B1/6 mice, C57B1/6 C3, and CR3-deficient mice, CD4 and CD8 ovalbumin T-cell receptor transgenic OT-I and OT-II mice, EO771/ovalbumin tumor model, RAM-MUC1 tumor model	Phagocytosis, binding, and staining assay, and qRT-PCR	Potent antitumor immune response, and drastic down-regulation of immunosuppressive cells, leading to the delayed tumor progression.	[87]
Mutated yeast β-glucan	6-week old pathogen free female BALB/C, C57BL/6, and CDF1 mice	Antitumor, immunostimulating, and NK cell activity	Dose-dependent inhibition of lung tumor metastasis via activation of macrophages and NK cells.	[88]

OL: *Omphalia lapidescens*; ICR mice: Imprinting control region mice; NMR: Nuclear magnetic resonance; GLC: Gas liquid chromatography; GLC-MS: Gas liquid chromatography-Mass spectroscopy; VDH: Vascular dilation and hemorrhage; MALDI-MS: Matrix-assisted laser desorption ionization-Mass spectroscopy; ELISA: Enzyme-linked immunosorbent assay; IAP: Immunosuppressive acidic protein; WGP: Whole glucan particles; MUC1: Mucine-1; mAb: Monoclonal antibody; BMDCs: Bone-marrow derived dendritic cells; IL: InterLeukin; DCs: Dendritic cells; EROD: Ethoxyresorufin Odeethylase; TNF-α: Tumor necrosis factor-α; NK cells: Natural killer cells; WGP: Whole glucan particles; qRT-PCR: Quantitative real-time-Polymerase chain reaction.

**Table 4:** Antitumor effects of β-glucans—human study.

β-Glucan	Cell Line	Analysis	Results	Reference
Fungal β-glucan	38–84 years old patients with advanced malignancies receiving chemotherapy	Changes in blood, and neutrophil counts, chemotherapy related symptoms (e.g., nausea and vomiting), and Hematological toxicity assay	Well tolerated in cancer patients receiving chemotherapy.	[129]
Yeast β-glucan	28–56 years old women with breast carcinoma	A randomized, double-blind, placebo-controlled study. Measurement of HRQL	Significant increase in global health status.	[130]

HRQL: Health-related quality of life.

**Table 5:** Immunomodulating effects of β-glucans—in vitro study.

β-Glucan	Cell Line	Analysis	Results	Reference
Yeast p-β-glucan (Cerevan)	Wistar rat thymocytes	HPGPC, Mitogenic, and co-mitogenic activity assay	Higher stimulation indices of immunomodulatory activity.	[90]
PGG-Glucan	Human monocytic cell lines U937, HL-60, THP-1, Murine monocytes J774.1, RAW264.7, P388D(I), Murine B cell line LB27.4, Primary human fibroblasts, Keratinocytes, Bronchial epithelial cells, Murine monocyte line BMC2.3, and T cell line DO11	Whole blood chemiluminescence assay, Microbicidal assay, Measurement of cytokine secretion from whole blood, ^3^H-PGG-Glucan binding assay, Flow cytometry, and Electrophoretic mobility shift assay	Induced activation of NF-κB-Like nuclear transcription factor in purified human neutrophils, and enhanced neutrophil anti-microbial function.	[131]
PS-G	DC from PBMC, and CD14^+^	Determination of cytokine levels, RT-PCR, Flow cytometry analysis, Western blot, Allogeneic MLR, EMS, and IKK activity assay	Increased activation and maturation of immature DC, suggesting a potential regulation of immune response.	[132]
Yeast p-β-glucan (synthetic glucan)	Porcine alveolar macrophages and bone hematopoietic cell-derived dendritic cells	MTT assay, ELISA, RACE PCR, and Phagocytic activity	Enhanced cell activity and phagocytosis, and complex collaborating interaction between dectin-1 and TLRs.	[133]
Barley β-glucan, Oat β-glucan, Fungal β-glucan	Human monocyte leukemia cell line	Size exclusion chromatography, Cytotoxicity assay, NO assay, H_2_O_2_ assay, Phagocytic activity, and qRT-PCR	Up-regulated inflammation related gene expression, and No production of NO, and H_2_O_2_.	[134]
Algal β-glucan	Murine splenic cells from BALB/c mice	NMR, Immunomodulatory activity assay, Immunofluorescence staining assay, and FACSCanto II flow cytometry	Increased activation of CD19^+^ B lymphocytes.	[135]
Polysaccharide glucan fractions	Spleen cells from female C3H/He mice, and Bone marrow cells from C57BL/6 mice	Mitogenic activity assay, and CSF-inducing activity assay	T-4-N and T-5-N fraction showed mitogenic and CSF-inducing activities.	[49]
Yeast β-glucan (WGP)	Mouse intestinal tumor cell line Colon26 produced in BALB/c mice	ELISA, and Tumor-protective effect assay	Stimulation of cytokines such as IL-2, IFN-γ, and TNF-α.	[136]
Bacterial β-glucan	Cancer cell lines, Human monocyte cell line, HPV-18-positive cervical cancer cell line, HPV-16-positive cervical cancer cell lines, such as CASki and C3, Hepatoma cancer cell line HepG2	RT-PCR, IFN-γ assay, NO, and cell viability assay	Synthesis of NO in the monocyte cell lines, enhanced cytotoxic, and antitumor activity.	[95]
Phycarine	Lewis lung carcinoma, and YAC-1 cell lines	Cytotoxicity assay, and Phagocytosis activity assay	Stimulation of both humoral and cellular branch of immune reactions could be used to cure gastrointestinal diseases.	[103]

HPGPC: High performance gel permeation chromatography; PS-G: Polysaccharide from *G. lucidum*; DC: Dendritic cell; PBMC: Peripheral blood mononuclear cells; CD: Cluster of differentiation; RT-PCR: Reverse transcription-Polymerase chain reaction; MLR: mixed leukocyte reaction; EMS assay: Electrophoretic mobility shift assay; IKK: Inhibitor of κB kinase; p: Particulate; MTT assay: Mitochondrial metabolic function assay; ELISA: Enzyme-linked immunosorbent assay; RACE: Rapid amplification of cDNA ends; TLRs: Toll like receptors; NO: Nitric oxide; H_2_O_2_: Hydrogen peroxide; qRT: Quantitative real-time; NMR: Nuclear magnetic resonance; CSF: colony stimulating factor; IL: InterLeukin; IFN-γ: Interferon-γ; TNF-α: Tumor necrosis factor-α.

**Table 6:** Immunomodulating effects of β-glucans—animal study.

β-Glucan	Organism	Analysis	Results	Reference
Polysaccharide glucan fractions	8–10 weeks old female C3H/He, C57BL/6, and ICR mice	Mitogenic activity assay, and CSF-inducing activity assay	T-4-N and T-5-N fractions showed mitogenic and CSF-inducing activities.	[49]
Fungal SSG glucan	CDF1 mice	Phagocytosis, H_2_O_2_, and CS activity assay	Enhanced colony stimulating activity, and activation of Peyer’s patch cells.	[137]
Yeast p-β-glucan	Male A/J, and Melanoma B16 model C57BL/6J mice	Histopathological analysis, and Bacterial susceptibility study	Significant reduction in the growth of a syngeneic anaplastic mammary carcinoma and melanoma B16. Prolonged survival of mice with subcutaneous tumor implants, decreased renal necrosis in *S. aureus* challenged mice and anti-staphylococcal activity.	[138]
Yeast β-glucan	Outbred male mice (CD-1, ICR), Inbred male rats (Fischer-344), Healthy mature and laboratory-conditioned cynomolgus male and female monkeys (*Macaca fascicularis*), seronegative to VEE virus-neutralizing antibody	Measurement of nonspecific potentiation, and specific enhancement of resistance	Significantly enhanced survival of mice challenged with either VEE virus or Rift Valley fever virus. Significant resistance of Glucan + VEE vaccine to homologous virus challenges.	[139]
Fungal Schizophyllan	3-weeks old specific-pathogen free male ICR/CRJ (CD-1) mice	Determination of protective effects of schizophyllan against primary Sendai virus infection in mice, and virus production in the infected lung and serum	Inhibited spread of virus in the lungs. Augmented protective immune responses induced by low doses of a live Sendai virus vaccine.	[140]
PGG + Cefazolin	Low inoculum albino Hartley guinea pigs	Bacterial growth, Prophylaxis studies, and MIC assay	PGG + Cefazolin synergistically prevented staphylococcal wound infection.	[141]
Oat β-glucan	6-weeks old female C57BL/6 mice	ELISA, and ELISPOT assay	Higher levels of total serum immunoglobulins and antigens against *Eimeria vermiformis* infection.	[142]
Yeast β-glucan (WGP)	6-weeks old female BALB/c mice	Anthrax-protective prophylactic effect and tumor-protective effect assay	Significant effect as a prophylactic treatment to reduce the mortality of anthrax infection.	[136]
SSG-glucan	6-week old, female inbred, specific pathogen-free NIH/OlaHsd mice	Mouse survival rate and the number of bacteria in blood samples	A significant dose-dependent effect of SSG against *Streptococcus pneumoniae* type 4 and 6B.	[143]
Fungal β-glucan	NC/Nga mice	Cell cytotoxicity, Sarcoma-180 tumor size, Blood IgE levels, Scratching index, and Human NK cell activity	Prolonged survival, reduction in tumor size, blood IgE levels, scratching index of NC/Nga mice, and enhanced cell cytotoxicity of human NK cells.	[144]
Yeast β-glucan	Male and female Wistar albino rats	Biochemical analysis, Apoptosis, Cell death, and Histopathological analysis	Reduced tissue damage. Inhibited the decrease in the stimulation index caused by methotrexate.	[145]
Bacterial β-glucan	4-weeks old male BALB/c and ICR mice	IFN-γ assay of PBMCs, and Antitumor activity assay	Induced IFN-γ and cytokines in spleens and thymus of mice. Enhanced cytotoxic and antitumor activity.	[95]
β-glucan from different sources	8-week old female BALB/c mice	Changes in blood glucose and blood cholesterol levels, and Phagocytosis of HEMA particles	Significant stimulation of IL-2 production and phagocytosis of peripheral blood leukocytes. Lowered blood sugar and cholesterol levels.	[6]
Phycarine	6–10 weeks old female BALB/c and C57B1/6J mice, and male and female pups	Apoptosis, Absorption, and Phagocytosis activity assay	Significant stimulation of phagocytosis, Strong influence on experimentally induced leucopenia, could be used to cure gastrointestinal diseases.	[103]
β-glucan from different sources	3-, and 8-weeks old BALB/c female mice	Phagocytosis, Cytokine assay, Tumor inhibition assay, and RT-PCR	Significant stimulation of phagocyte activity. Increase synthesis and release of ILs, and TNF-α. Inhibited growth of tumor cells in breast cancer cells.	[146]
Yeast insoluble-β-glucan	8-weeks old female BALB/c mice	Phagocytosis, Cold stress response, Changes in serum corticosterone and cytokine production levels	Inhibition of stress related suppression, normal phagocytosis activity. Inhibition of corticosterone, above normal levels of IL-6 and IL-12 secretion.	[147]
Lentinan	Male BN/RijHsd rats	Hematopoiesis, Flow cytometry, and Serum cytokine analysis	Significant increase in weight gains, monocytes, blood cells, circulatory cytotoxic T-cells and a reduction in anti-inflammatory cytokines IL-4, IL-6, and IL-10. Increased in cage-side health of acute myeloid leukemia.	[148]
Polysaccharide β-glucan	6–8 weeks old male Swiss albino mice	Macrophage activity assay, Flow cytometry, In vitro NK cell assay, Serum biochemistry and Histological analysis	Significant increase in IL-1 and NO production and increased phagocytic potential. Increased activation of NK cells and proliferation of splenocytes.	[149]
Paramylon	5-week old NC/Nga mice	Histopathological, and Macroscopic analysis	Significantly inhibited the development of atopic dermatitis-like skin lesions with no adverse effect on weight loss.	[150]
β-glucan	5–6 week old Sprague-Dawley male and female rats	Subacute toxicological study, Clinical examination, Pathological analysis, and Flow cytometry	Significant increase in red blood cell, white blood cell, hemoglobin, and thrombocytes. No adverse effect on general condition, growth, behavior, and feed consumption.	[151]
Commercial β-1,3;1,6-glucan	Private owned dogs with signs of atopic dermatitis, the dog breeds include: West highland white terriers, Staffordshire bull terriers, German shepherds, Heidewachtels small Munsterlander pointers, Crossbreeds and others	Signs of itching, How many times dog scratches, and Changes in skin color, and thickness	Canine atopic dermatitis diminished.	[152]
β-glucan	Adult male Sprague Dawley rats	Physical exercise, Determination of exhaustive time, and Immunohistochemical analysis of oncogenes (*c-Jun* and *c-Fos*)	An alleviating effect on the exercise-induced stress through the suppression of oncogenes expression in the brains of exhausted rats.	[153]

ICR mice: Imprinting control region mice; CSF: Colony stimulating factor; H_2_O_2_: Hydrogen peroxide; CS activity: Colony stimulating activity; VEE: Venezuelan equine encephalitis; MIC: Minimum inhibitory concentration; ELISA: Enzyme-linked immunosorbent assay; ELISPOT: Enzyme-linked immunosorbent spot assay; IgE: Immunoglobulin E; NK: Natural killer; IFN-γ: Interferon-γ; PBMC: Peripheral blood mononuclear cells; HEMA: Hydroxyethylmethacrylate; IL: InterLeukin; RT-PCR: Reverse transcription-Polymerase chain reaction; NO: Nitric oxide.

**Table 7:** Immunomodulating effects of β-glucans—human study.

β-Glucan	Organism	Analysis	Results	Reference
PGG-glucan	More than 18 years old patients who underwent a major abdominal or non-cardiac thoracic surgery	Postoperative infection response	A dose-dependent protective response against the postoperative infection.	[154]
β-1,3-polyglucose (β-glucan)	*Paracoccidiodes brasiliensis* infected patients	Erythrocyte sedimentation rate, and Phytohemagglutinin skin test	Increase in number of CD^4+^ T lymphocytes, higher serum level of TNF-α. Stronger and more favorable response to therapy.	[155]
Commercial Curdlan, Paramylon, Laminarin, Scleroglucan, Pustulan	28–56 years old, healthy as well as volunteer patients allergic to house dust mites	Histamine release test from blood leukocytes	Enhanced IgE-mediated histamine release.	[156]
Yeast β-glucan	6–12 years old children with mild to moderate persistent asthma	Calculation of serum IL-10, and Asthmatic symptoms	Significant increase in serum IL-10 levels and a significant reduction in asthma.	[157]
Oat β-glucan	Healthy, normal female and male volunteers, with mean age: 22.6 ± 0.7 years	Changes in blood plasma glucose, insulin, ghrelin, CCK, PYY, and GLP-1 levels. Subjective appetite measurements, and Biochemical analysis	Postprandial increase in satiety, plasma glucose, insulin, CCK, GLA-1, and PYY and a greater decrease in postprandial ghrelin.	[158]
WGP-glucan	Male and female volunteers	Flow cytometry, and Separate multiplex assay	A significantly enhanced CD14^+^, and CD14^+^/CD16^+^. LPS-stimulated production of IFN-γ and IL-2, IL-4, and IL-5.	[159]
Fungal β-glucan	Clinical pulmonary disease and trauma, suffering patients	Serum lipid profile analysis, Serum hs-CRP, cytokine, and NK cell activity assay	Increased NK cell activities, and serum pre-albumin, and decreased hs-CRP.	[160]
Yeast β-glucan (Wellmune, WGP)	18–53 years old, male and female marathon runners	A randomized, double-blind, placebo-controlled trial. Profile of mood state assessment	Decreased URTI symptoms, fatigue and anger. An increase in overall health and vigor.	[161]
Yeast β-glucan (Wellmune, WGP)	18–65 years old, moderate to high-stressed male and female adults	A randomized, double-blind, placebo-controlled trial. Respiratory tract infection analysis	Decreased URTI symptoms, fatigue and tension. Improved overall health and vigor.	[162]
Yeast β-glucan (Wellmune, WGP)	26–50 years old healthy women with moderate levels of psychological stress	A randomized, double-blind, placebo-controlled trial. Changes in mental/physical energy levels and mood states.	Decreased URTI symptoms, and increased mental/physical energy levels.	[163]
Yeast β-Glucan (Glucan #300)	8–12 years old, male and female children with chronic respiratory problems	A randomized, double-blind, placebo-controlled trial. Changes in levels of lysozyme, albumin, and CRP in saliva	Increased changes in production of lysozyme and CRP. Improvement in the general condition and stimulated mucosal immunity.	[164]
Yeast β-glucan (Glucan #300)	8–12 years old children with chronic respiratory problems	A randomized, double-blind, placebo-controlled trial. Measurement of levels of IgA, IgG, and IgM	A significant increase in production of salivary immunoglobulins, and improvement in the mucosal immunity.	[165]
Yeast β-glucan (Glucan #300)	8–12 years old children with chronic respiratory problems	A randomized, double-blind, placebo-controlled trial. Physical endurance test and estimation of eNO levels	A significant improvement in physical endurance, eNO levels, and general conditions.	[166]
Yeast β-glucan (Glucan #300)	8–12 years old children with chronic respiratory problems	A randomized, double-blind, placebo-controlled trial. Measurement of levels of lysozyme, albumin, CRP, and calprotectin in saliva	A significant increase in production of salivary CRP, lysozyme, and calprotectin.	[167]
Yeast β-glucan (Glucan #300)	7–14 years old children with chronic respiratory problems	A randomized, double-blind, placebo-controlled trial. Measurement of levels of cortisol, salivary IgE, and cotinine	Decreased salivary cortisol and cotinine levels. An increase in physical endurance and improvement of affected children.	[168]
Yeast β-glucan (Glucan #300)	8.2–12.4 years old children with chronic respiratory problems	A randomized, double-blind, placebo-controlled trial. Measurement of levels of eNO, salivary IgA, and physical activity (6MWT test)	A significant decrease in eNO levels. Physical endurance and stabilization of the salivary IgA levels.	[169]
Imunoglukan P4H (a syrup containing Pleuran)	3–7 years old children with RRTIs	Open-label trial. Monitoring the occurrence of RRTIs	A 50% reduction in frequency of RRTIs.	[170]
Imunoglukan P4H (a syrup containing Pleuran)	3–8 years old children with RRTIs	A randomized, double-blind, placebo-controlled trial. Blood sample analysis for immune parameters	Significant reduction in frequency of RRTIs, number of flu-like diseases, respiratory tract infections, and an increase in number of healthy children.	[171]
Imunoglukan P4H (a syrup containing Pleuran)	2–5, and 6–10 years old children with RRTIs	A randomized, double-blind, placebo-controlled trial. Measurement of total IgE, specific IgE levels, and BECs	Significant reduction of peripheral blood eosinophilia as well as stabilized levels of total IgE in serum.	[172]
The effect of Imunoglukan P4H (a cream containing Pleuran)	Male and female patients with atopic dermatitis, with mean age of 20.4 years	Objective and subjective symptoms of AD, including visual analysis, EASI	Significant decline in the number of days with AD exacerbation and its severity. Decline of pruritus by visual analog scale. Significant decline of EASI on the site of β-glucan application.	[173]
Imunoglukan P4H (a syrup containing Pleuran)	3 years old children with RRTIs	A multi-center, open-label trail. Monitoring the occurrence of RRTIs	A significant reduction in RRTIs frequency, and the occurrence of respiratory diseases, such as common cold, laryngitis, tonsillpharyngitis, pneumonia, and bronchitis.	[174]
Imunoglukan P4H (a syrup containing Pleuran)	3.7 years old children with RRTIs	Open-label trail. Monitoring the occurrence of RRTIs	A significant reduction in RRTIs frequency, and the occurrence of respiratory diseases, such as laryngitis, common cold, and bronchitis.	[175]

CD: Cluster of differentiation; TNF-α: Tumor necrosis factor-α; IgE: Immunoglobulin E; IL: InterLeukin; CCK: Cholecystokinin; PYY: Peptide YY; GLA: Glucagon-like peptide-1; LPS: lipopolysaccharide; IFN-γ: Interferon-γ; hs-CRP: high-sensitivity C-reactive protein; NK: Natural killer; URTI: Upper respiratory tract infection; CRP: C-reactive protein; IgA: Immunoglobulin A; IgG: Immunoglobulin G; IgM: Immunoglobulin M; eNO: Exhaled nitric oxide; 6MWT: 6-min walking test; RRTIs: Recurrent respiratory infections; BECs: Blood eosinophil cell counts; AD: Atopic dermatitis; EASI: Eczema area and severity index.

**Table 8:** Bone regeneration/bone injury healing effects of β-glucans—in vitro study.

β-Glucan	Cell line	Analysis	Results	Reference
PGG-glucan	Human BMMC, and isolated bone marrow CD34^+^ cells	BMMC myeloid colony formation assay, Human hematopoietic activity, and ELISA	Increased BMMC myeloid colony formation, and enhanced human hematopoietic activity.	[176]
Polycalcium [Polycan and calcium lactate-gluconate (1:9)]	Human hOBs, and murine osteoclast progenitor (RAW264.7) cells	Cell proliferation and alkaline phosphatase activities of osteoblasts and osteoclast differentiation	Stimulation of osteoblast proliferation and prevented RANKL-induced osteoclast differentiation. Accelerated bone formation and inhibited bone resorption activity.	[177]
Fungal β-glucan	Normal diploid human fetal dermal fibroblast cell line (FW20-2), and primary human dermal fibroblasts	Cell proliferation assay, RP-HPLC, Fibroblast-populated collagen lattice, and wounding	Reduction in fibroblast proliferation and migration were significantly and dose-dependently inhibited.	[178]
Chitosan/β-1,3-glucan/hydroxyapatite complex (Chit/glu/HA)	Human fetal osteoblast cell line (hFOB 1.19)	Biocompatibility of scaffolds, cytotoxicity, and osteoblast proliferation rate, Porosity using computed microtomography analysis and mechanical properties by compression test	Improved flexibility and porosity, significant higher water uptake capability, favorable osteoblast survival, proliferation, and spreading, but poor mechanical properties.	[179]

BMMC: Bone marrow mononuclear cells; CD: Cluster of differentiation; ELISA: Enzyme-linked immunosorbent assay; hOBs: Human primary osteoblasts; RANKL: Receptor activator of nuclear factor ligand; RP-HPLC: Reverse phase-High performance liquid chromatography.

**Table 9:** Bone regeneration/bone injury healing effects of β-glucans—animal study.

β-Glucan	Organism	Analysis	Results	Reference
β-glucan	2–3 months old CD-1 male mice	Chromosomal aberrations and mitotic activity	Reduced total number of cells with structural chromosomal aberrations in bone marrow and spermatogonial cells. Markedly restored mitotic activity of bone marrow cells, suppressed by anti-neoplastic drugs.	[180]
Polycalcium [Polycan and calcium lactate-gluconate (1:9)]	6-weeks old, Sprague-Dawley specific pathogen-free female ovariectomy-induced osteoporotic rats	Changes in body and bone weight, serum osteocalcium and bone-specific alkaline phosphatase levels, Urine Dpd/creatinine ratio, and Histological analysis	Markedly decreased OVX-induced osteoporotic changes. Preserved bone mass and strength.	[181]
Polycalcium [Polycan and calcium lactate-gluconate (1:9)]	6-weeks old Sprague-Dawley specific pathogen-free male rats	Changes in body weight, knee thinness, cartilage glycosaminoglycan content, and Histopathological assay	Inhibited osteoarthritis related changes and induction of chondrocyte proliferation.	[182]
Polycal [Polycan and calcium-gluconate (2:98)]	6-weeks old male SD (Crl:CD1) rats	Changes in body weight, alveolar bone loss index, total number of buccal gingival aerobic bacterial cells, IL-1, TNF-α levels, and myeloperoxidase activity	Bacterial proliferation, periodontitis, and alveolar bone loss induced by ligature placement were significantly inhibited.	[183]
CHAP + β-glucan composite material	6-months old New Zealand male white rabbits	Radiological imaging and Histological analysis. Peripheral quantitative computed tomography, Densitometry and SEM analysis	No sign of graft rejection, stimulating effect of biomaterial on bone formation and mineralization. Enabled regeneration of bone tissue.	[184]
Polycan	An oestrogen-deficient ovariectomy model and a hypocalcemic and hypoparathyroid thyroparathyroidectomy model	Changes in bone mineral density in the femur, tibia, and lumber (L6) vertebrate using dual-energy X-ray absorptiometry, and changes in Ca bioavailability	Marked increase in the BMD of femur, tibia, and L6. Enhanced absorption and bioavailability of Ca and improved Ca balance.	[76]
Polycan	6-weeks old virgin Sprague-Dawley pathogen free female rats as an oestrogen-deficient ovariectomy model	Changes in body weight, bone mineral content, density, failure load, Histological profile, and Histomorphometric indices	Inhibited OVX-induced alterations in bone resorption. Increased serum expression levels of BLAP and all histomorphometrical indices for bone formation.	[77]

CD: Cluster of differentiation; Dpd: Deoxypyridinoline; OVX: Ovariectomy; IL: InterLeukin; TNF-α: Tumor necrosis factor-α; SEM: Scanning electron microscopy. CHAP: Carbonated hydroxyapatite; BMD: Bone mineral density; Ca: Calcium; BALP: Bone-specific alkaline phosphatase.

**Table 10:** Bone regeneration/bone injury healing effects of β-glucans—human study.

β-Glucan	Organism	Analysis	Results	Reference
Polycalcium (Polycan + calcium lactate-gluconate)	40–60 years old healthy women	Anti-osteoporotic effect, Measurement of changes in DPYR, OSC, BALP, CTx, and P levels	Improved bone metabolism and well tolerated polycalcium effect.	[185]
Polycan	40–70 years old, healthy premenopausal women	Anti-osteoporotic effect, Measurement of changes in OCS, BALP, Ca, and P levels	Increased changes in OSC, and BALP, Ca, P, CTx, NTx, and DPYR. Increase in CTx was modestly inhibited.	[186]

DPYR: Deoxypyridinoline; OSC: Osteocalcium; BALP: Bone-specific alkaline phosphatase; Ca: Calcium; P: Phosphorus; CTx: C-telopeptide of collagen cross-links; NTx: N-telopeptide of collagen cross-link.

**Table 11:** Anti-diabetic/anti-obesity effects of β-glucans—animal study.

β-Glucan	Organism	Analysis	Results	Reference
Lentinan	Female BALB/c mice	Spectrophotometric analysis of the total CYP contents, Western blot analysis, ECOD, EROD, and EMSA activities	Suppression of constitutive and 3-methylcholanthrene-induced CYP expression and EROD activity in liver.	[187]
Chitin-glucan	9-weeks old, male C57BL6/J mice	Oral glucose tolerance test, Microbial analysis of the cecal contents, ELISA, and Histochemical analysis	Decreased mouse gut microbiota, body weight gains, fat mass development, glucose intolerance, hepatic triglyceride accumulation and hypercholesterolemia.	[188]
Polycan	7-weeks old male hamsters	Changes in body weight, food consumption, liver weight, Serum biochemistry, Histopathological, and Histomorphometric analysis	No significant change in body weight and food consumption, serum levels of AST, ALT, triglyceride, LDL- and total-cholesterol levels. Dose-dependent reduction of atherosclerosis with relatively good protective effects on liver damage.	[75]
Yeast β-glucan + *Folium mori* extract (BG-FM)	STZ-induced diabetic rats	Changes in blood glucose levels, body weight, liver, and kidney weight, and Serum BUN, AST, ALT levels	Reduced hyperglycemic changes in the *F. mori* extract. Dose-dependent increase in anti-diabetic and hypoglycemic effect.	[189]

CYP: Cytochrome; ECOD: Ethoxycoumarin-O-deethylation; EROD: Ethoxyresorufin-O-deethylation; EMSA: Electrophoretic mobility shift assay; ELISA: Enzyme-linked immunosorbent assay; AST: Aspartate aminotransferase; ALT: Alanine aminotransferase; LDL: Low-density lipoprotein; STZ: Streptozotocin; BUN: Blood urea nitrogen.

**Table 12:** Anti-diabetic/anti-obesity effects of β-glucans—human study.

β-Glucan	Organism	Analysis	Results	Reference
Oat β-glucan	49–57 years old NIDDM men and women, with a BMI range of 22.6–38.9 kg/m^2^	Plasma glucose and glycemic response	Increased plasma glucose, postprandial insulin, and 50% decrease in glycemic response.	[190]
Barley β-glucan	26–30 years old, healthy men with mildly higher fasting total cholesterol concentration, with a BMI range of 22–25 or 27–29 kg/m^2^	Insulin, glucose, cholecystokinin, and lipid response	Increased plasma glucose and insulin concentrations, stimulation of reverse cholesterol transport contributing to the cholesterol lowering ability.	[191]
Barley β-glucan	20–27 years old, healthy, non-diabetic men and women	Sensory properties, proximate composition, and glycemic indices	Dose-dependent decrease in glycemic content, and decreased postprandial glycemic index.	[192]
Oat β-glucan	59–63 years old, type 2 diabetic men and women, with a BMI range of 27–31 kg/m^2^	Changes in blood glucose, total-, HDL-, and LDL-cholesterol, and triglyceride levels	Reduced blood glucose levels, glycemic indices, and postprandial glycemia.	[193]
Oat β-glucan	61–73 years old, type 2 diabetic men and women, with a BMI range of 25.4–32.4 kg/m^2^	Glucose tolerance test, and Finger-prick capillary blood analysis	Decreased glycemic, and postprandial glycemic response.	[194]
Oat β-glucan, Barley β-glucan	18–70 years old, healthy men and women with mildly elevated serum cholesterol concentration and a BMI range of 20–30 kg/m^2^	Changes in plasma glucose, serum total-, HDL-, and LDL-cholesterol, triacylglycerol, apolipoproteins A1, and postprandial changes in serum	Oat β-glucan showed reduced total-cholesterol, postprandial glucose, and insulin concentrations as well as improved lipid and glucose metabolism.	[195]
Oat β-glucan	Healthy volunteer men and women	Changes in insulin and glycemic response index, and Glucose tolerance test	Reduced insulin and glycemic index.	[196]
Oat β-glucan	Healthy volunteer men and women	Blood insulin and glucose response	Significant reduction in insulin and glucose responses in healthy people.	[197]
Barley β-glucan	26–50 years old, healthy men and women, with a BMI range of <30 kg/m^2^	Changes in blood glucose contents, and GR, and GI response	Significantly reduced postprandial blood glucose, and glycemic index.	[198]
Oat β-glucan	30–75 years old, diabetic men and women, with a BMI range of 20–35 kg/m^2^	Changes in lipid profile, apo B, TAG, HbA1c, and fasting glucose concentrations	A single daily ingestion of 3.5 g oat β-glucan showed no significant changes in lipid profile and HbA1c in type 2 diabetic subjects whereas, TAG significantly decreased.	[199]

NIDDM: Non-insulin dependent diabetes mellitus; BMI: Body mass index; HPLC: High performance liquid chromatography; HDL: High-density lipoprotein; LDL: Low-density lipoprotein; GR: Glycemic response; GI: Glycemic index; TAG: Triacylglycerol; HbA1c: Glycosylated hemoglobin.

**Table 13:** Cholesterol and blood lowering effects of β-glucans—animal study.

β-Glucan	Organism	Analysis	Results	Reference
Yeast-WGP	8-week old hypercholesterolemic BALB/c mice	Phagocytosis, and Biochemical analysis	A dose-dependent decrease in plasma cholesterol and triglyceride levels.	[200]
Yeast β-glucan	Sprague-Dawley rats	Serum total cholesterol, triglyceride, and malondialdehyde analysis	Significantly reduced and maintained cholesterol levels in blood plasma and liver. Triglyceride and MDA levels significantly reduced.	[201]

WGP: Whole glucan particle; MDA: Malondialdehyde.

**Table 14:** Cholesterol and blood lowering effects of β-glucans—human study.

β-Glucan	Organism	Analysis	Results	Reference
Oat β-glucan	30–65 years old men and women with LDL-cholesterol levels of >3.37 mmol/L	Changes in total-, LDL-, and HDL-cholesterol levels	Significantly decreased total-, and LDL-cholesterol concentrations.	[202]
Barley β-glucan	21–42 years old healthy men with total-cholesterol levels (between 4.1 and 6.2 mmol/L), triacylglycerol levels (<2.26 mmol/L), and a BMI range of 22–25 or 27–29 kg/m^2^	Changes in plasma glucose, insulin, triacylglycerol, cholesterol concentrations, and Radioimmunoassay	Increased plasma glucose, insulin, triacylglycerol and cholecystokinin levels. Stimulation of reverse cholesterol transport mechanism.	[191]
Yeast β-glucan	20–60 years old hypercholesterolemic obese male patients with serum total cholesterol concentrations of >6.21 mmol/L	Changes in plasma total-, LDL-, and HDL- cholesterol and triacylglycerol levels	Reduced plasma total-, HDL- and LDL-cholesterol concentrations. Triacylglycerol concentrations did not change significantly.	[203]
Oat β-glucan	30–70 years old mild-to-moderate hyperlipidemic healthy men and women, with a BMI range of 20–32 kg/m^2^	Changes in total-, LDL-, and HDL-cholesterol, triacylglycerol, glucose, insulin, postprandial triacylglycerol, glucose, and insulin concentrations	No significant difference in total-, or LDL-cholesterol at a low dosage of β-glucan (3 g/d).	[204]
Oat β-glucan	33–82 years old hyperlipidemic men and women	Changes in blood lipids, apolipoproteins, cardiovascular risk factor, blood pressure, and gastrointestinal symptoms	Reduced total-, total- to HDL-cholesterol ratio, LDL- to HDL-cholesterol ratio. Apolipoprotein (B:A-I) reduction in CVD risk, and small reduction in blood pressure.	[205]
Barley β-glucan	18–65 years old mildly hyperlipidemic men, with a BMI range of 22–32 kg/m^2^	Changes in total-, LDL-, and HDL-cholesterol, triacylglycerol, fasting plasma glucose, and postprandial plasma glucose levels	No significant change in total-, LDL- or HDL-cholesterol, triacylglycerol, fasting glucose, or postprandial glucose.	[206]
Oat β-glucan	18–65 years old mildly hypercholesterolemic men and women, with a BMI range of >30 kg/m^2^	Changes in total-, HDL-, LDL- cholesterol and triacylglycerol levels. High performance size-exclusion chromatography	Decreased LDL-, and ratio of total- to HDL-cholesterol concentrations. No significant change in HDL-cholesterol and triacylglycerol levels.	[207]
Barley β-glucan	28–62 years old moderately hypercholesterolemic men	Changes in total-, HDL-, and LDL-cholesterol, and triacylglycerol concentrations, and NMR	Significantly lowered triacylglycerols, total-, and LDL-cholesterol, but higher HDL-cholesterol concentrations.	[25]
Barley β-glucan	38–53 years old mildly hypercholesterolemic men and women with a BMI range of 25–37 kg/m^2^	Changes in cholesterol, and triacylglycerol levels, and NMR	Lowered total-, and HDL-cholesterol concentrations. Triacylglycerol concentration did not differ.	[26]
Oat β-glucan	30–65 years old men and women with elevated blood pressure or stage-1 hypertension	Changes in plasma glucose, insulin levels, and blood pressure	Lowered systolic and diastolic blood pressure.	[208]
Oat β-glucan	More than 40 years old men and women with elevated blood pressure (between 130 and 179 mm Hg), controlled with anti-hypertensive medications	Clinical laboratory measurements of plasma glucose and insulin levels, Oxidative stress, and Blood pressure	Lowered insulin levels, and systolic and diastolic blood pressures. Biomarkers of oxidative stress did not show significant differences.	[209]
Oat β-glucan	22–65 years old hypercholesterolemic men and women at a risk for CVD	Changes in total-, HDL-, and LDL-cholesterol, triglycerides, glucose, insulin, homocysteine, and CRP levels, and blood pressure	Significant reduction in total-, LDL-cholesterol in subjects with elevated cholesterol levels, and a significant reduction of lipids.	[109]
Barley β-glucan	30–60 years old hypercholesterolemic Japanese men with a BMI range of >22 kg/m^2^	CT-scan, Blood analysis for serum TG, TC, LDL-, and HDL-cholesterol levels	Significant reduction in serum concentration of LDL-C, TC, and visceral fat area.	[210]
Oat β-glucan	50–75 years old patients (both men and women) with T2D, LDL-cholesterol concentration (>3.37 mmol/L), and a BMI range of 23–35 kg/m^2^	Changes in BMI, waist circumference, LDL-, Total-, HDL-, and non-HDL-cholesterol concentrations, HbA, and systolic BP	Significant reduction in LDL-, total-cholesterol concentrations, FPI, and Homa-IR. Improvement in lipid profile and insulin resistance in patients with T2D.	[27]

LDL: Low-density lipoprotein; HDL: High-density lipoprotein; BMI: Body mass index; CVD: Cardiovascular disease; NMR: Nuclear magnetic resonance; CRP: C-reactive protein; CT-scan: Computerized axial tomography; TG: triglycerides; TC: Total cholesterol; T2D: Type 2 diabetes; BP: Blood pressure; FPI: Fasting plasma insulin; Homa-IR: Homeostasis model assessment-insulin resistance.

**Table 15:** Antigenotoxicity/antimutagenicity/antioxidative effects of β-glucans—in vitro study.

β-Glucan	Cell line	Analysis	Results	Reference
Yeast β-glucan, Fungal β-glucan + chitin complex from *Aspergillus niger*	Chinese hamster lung fibroblasts V79	HPLC, H_2_O_2_ assay, and Comet assay	Increased comet activity, and protective effect against oxidative DNA damage.	[211]
Yeats cell wall mannan, and mannan conjugates	Unicellular flagellate *Euglena gracilis* cells exposed to the genotoxic agents ofloxacin and acridine orange	HPLC, FT-IR spectroscopy, Antioxidant assay (ABTS-radical scavenging activity), and *Euglena gracilis* mutagenicity assay	Protective antigenotoxic activity, and inhibited AO-induced chloroplast DNA damage.	[212]
β-glucan	Chinese hamster ovary cell line, and the hepatoma cell lines from *Ratus vovergicus*	Micronucleus assay	Increased chemoprotective, and anti-mutagenic activity.	[213]
Fungal β-glucan	Human peripheral lymphocytes	Binding, Comet assay, and H_2_O_2_ assay	Dose-dependent protective effect against damage induced by H_2_O_2_ and Trp-P-2.	[214]
Barley β-glucan	Chinese hamster ovary cell line, and the hepatoma cell line	Chromosomal aberration assay, and Anti-clastogenic activity	Protective effect in the presence of a DNA polymerase-β inhibitor.	[215]
Fungal β-glucan	Human hepatoma cell line	FT-IR, NMR, Comet assay, and Cytokinesis-block micronucleus assay	Does not exert a genotoxic or mutagenic effect, but protected effect against DNA damage caused by bezo[a]pyrene (B[a]P).	[216]
Chrysolaminarin	-	FT-IT, NMR, H_2_O_2_, and DPPH-radical scavenging activity	Significant hydroxyl radical scavenging activity.	[101]
Fungal β-glucan (SBG)	Human umbilical vein endothelial cells, highly metastatic B16-F10 and B16-BL6 cells	Dorsal air sac assay, Matrigel plug assay, and Methylation analysis	Suppression of growth and number of metastatic tumor foci in lung, and improved anti-angiogenic and anti-metastatic effect.	[74]

HPLC: High performance liquid chromatography; H_2_O_2_: Hydrogen peroxide; DNA: Deoxyribonucleic acid; FT-IR: Fourier transform infrared spectra; AO: Acridine orange; NMR: Nuclear magnetic resonance.

**Table 16:** Antigenotoxicity/antimutagenicity/antioxidative effects of β-glucans—animal study.

β-Glucan	Organism	Analysis	Results	Reference
Fungal β-glucan (SBG)	Neoplasm, Female ICR, and C57BL/6J mice	Dorsal air sac assay, Matrigel plug assay, and Methylation analysis	Suppression of growth and number of metastatic tumor foci in lung. Anti-angiogenic and anti-metastatic effect.	[74]
β-glucan	Wistar albino rats	SOD, MPO, MDA, LPO, and GSH activity analyses	A significant reduction in AST, ALT, LDH, GGT, MPO, LPO, and MDA levels and greater levels of GSH and SOD.	[217]
Yeast β-glucan	Male Wistar albino young, healthy rats	Antioxidant activities (SOD, GSH-Px, CAT, MDA)	Significantly reversed elevation of MDA levels and reduction in SOD activities. Slightly enhanced activity of CAT and prevented depletion of GSH-Px activity caused by EMR, and higher antioxidant activities.	[218]
Fungal β-glucan	6–8 weeks old swiss albino mice	Antioxidant activities (H_2_O_2_, Ferric reducing power assay), lipid peroxidation assay, Biochemical and Hematological analyses	Increased post-irradiation survival of mouse, significant reduction in number of aberrant cells.	[219]

ICR: Imprinting control region; SOD: Superoxide dismutase; MPO: Myeloperoxidase; MDA: Malondialdehyde; LPO: Lipid peroxide; GSH: Glutathione; AST: Aspartate aminotransferase; ALT: Alanine aminotransferase; GGT: Gamma glutamyl transpeptidase; CAT: Catalase; EMR: Electromagnetic radiation; H_2_O_2_: Hydrogen peroxide.
